# miR-129-5p Inhibits Bone Formation Through TCF4

**DOI:** 10.3389/fcell.2020.600641

**Published:** 2020-11-06

**Authors:** Chong Yin, Ye Tian, Yang Yu, Chaofei Yang, Peihong Su, Yipu Zhao, Xue Wang, Kewen Zhang, Jiawei Pei, Dijie Li, Zhihao Chen, Yan Zhang, Zhiping Miao, Airong Qian

**Affiliations:** ^1^Lab for Bone Metabolism, Xi’an Key Laboratory of Special Medicine and Health Engineering, Key Lab for Space Biosciences and Biotechnology, Research Center for Special Medicine and Health Systems Engineering, NPU-UAB Joint Laboratory for Bone Metabolism, School of Life Sciences, Northwestern Polytechnical University, Xi’an, China; ^2^Tianjin Key Laboratory on Technologies Enabling Development Clinical Therapeutics and Diagnostics (Theranostics), School of pharmacy, Tianjin Medical University, Tianjin, China

**Keywords:** osteoporosis, osteoblast, miR-129-5p, TCF4, recombinant RNA

## Abstract

Osteoporosis is a frequently occurring bone disease in middle-aged and aged men and women. However, current therapies on this disease are still not ideal. MicroRNAs (miRNAs) are a class of endogenous non-protein-coding RNA with a length of 18–25 nucleotides. miRNAs have been identified as important regulators for development, metabolism, carcinogenesis, and bone formation. miR-129-5p has been reported as a regulator of cancer and neuroscience, whereas studies about its function on bone formation is still limited. In this study, we investigated the function and mechanism of miR-129-5p on osteoblast differentiation and bone formation. We have assessed the expression of miRNAs in bone mesenchymal stem cells from aging and menopause osteoporosis C57BL6 mice. The expression of miR-129-5p was altered in all osteoporosis models. Besides, the expression of miR-129-5p was negatively correlated with osteoblastic differentiation markers in the femur tissues of C57BL/6 mice of different ages. We further demonstrated that overexpression of miR-129-5p inhibited osteoblast differentiation in MC3T3-E1 cell line, as well as bone formation of C57BL/6 mice. On the other hand, down-regulation of miR-129-5p enhanced osteoblast differentiation and bone formation. We also found that miR-129-5p inhibited Wnt/β-catenin pathway in osteoblast. The target gene of miR-129-5p has been forecasted and proved as *Tcf4*. We further found that plasmid containing *Tcf4–*3′ UTR sequence enhanced osteoblast differentiation, as well as Wnt/β-catenin pathway in MC3T3-E1 cells. To further investigate the rescue effect of miR-129-5p inhibitor, we manufactured bioengineered novel recombinant miR-129-5p inhibitor through *Escherichia coli* system and then tested its function. The results showed that the novel recombinant miR-129-5p inhibitor promoted osteoblast differentiation and greatly ameliorated menopause osteoporosis in C57BL6 mice. In conclusion, we have discovered miR-129-5p as an inhibitor of bone formation. miR-129-5p inhibited downstream transcription factors of Wnt/β-catenin pathway through targeting *Tcf4*. Moreover, novel recombinant miR-129-5p inhibitor showed rescue effect on osteoporosis. This study has revealed a new mechanism of osteogenic differentiation and provided novel therapeutic strategies for treatment of skeletal disorders.

## Introduction

Osteoporosis is a high-incident bone disease in middle-aged and aged men and women, with symptoms identified as declined bone mass and bone strength, deteriorated bone microarchitecture, and increased risk of fracture (Hendrickx et al., 2015). The causes of osteoporosis are multiple and complex. Among those, declined osteoblast differentiation, which leads to discouraged bone formation, was proved as one of the major causes of osteoporosis (Jabbar et al., 2011; Gennari et al., 2016). Osteoblast differentiation could be affected by multiple signaling pathways and osteogenic factors, including microRNAs (miRNAs), which were proved to be highly correlated with osteoblast differentiation and osteoporosis.

MicroRNAs are a class of endogenous non-protein-coding RNA with 18–25 nucleotides in length. miRNAs have been known as important regulators for development, metabolism, carcinogenesis, and bone formation (Bartel, 2009; Rigoutsos and Furnari, 2010; Chen et al., 2017). Emerging numbers of studies have reported miRNAs as regulators for osteoblast differentiation and bone formation, such as miR-21, miR-214, miR-188, miR-148-3p, miR-422a, etc. (Wang et al., 2013; Cao et al., 2014; Li et al., 2015, 2017; Yuan et al., 2019). These studies suggested that the functional and mechanism researches on osteogenic miRNAs would be helpful to develop potential therapeutic strategies for osteoporosis. miR-129-5p has been reported as a regulator of cancer and neural disease (Li G. et al., 2019; Zeng et al., 2019), whereas studies on its function in bone formation are relatively limited. Shi et al. (2020) reported that hsa-miR-129-5p inhibited osteogenic differentiation of adipose-derived stem cells via Wnt/β-catenin pathway, which implied that miR-129-5p may also be an inhibitor of bone formation. Therefore, the reduction of miR-129-5p level in bone tissue might be a potential anabolic strategy for ameliorating osteoporosis.

Biological approaches have been made to use live cells to bioengineer natural RNA molecules that are ready to use as RNAi agents (Ponchon et al., 2009; Huang et al., 2013; Chen et al., 2015; Ho and Yu, 2016). These recombinant RNA technologies provided a novel way for fast production of large quantities of chimeric RNAs in a cost-effective manner. As benefit from this technological progress, we used an improved ncRNA carrier (nCAR; Ho et al., 2018) for production of recombinant miR-129-5p inhibitor (nCAR/anti129). This bioengineered nCAR/anti129 may better capture the activity of natural RNAs and thus have greater potential for clinical application.

In this study, we have identified miR-129-5p as an inhibitor for osteoblast differentiation and bone formation. We found that miR-129-5p blocked downstream transcript factors of Wnt/β-catenin pathway by targeting *Tcf4*. In addition, novel recombinant miR-129-5p inhibitor that was manufactured through *Escherichia coli* system was applied to *in vivo* study to further figure out its rescue effect on postmenopausal osteoporosis. The study has discovered a novel mechanism regulating osteoblast differentiation and bone formation and provided new ideas for the translational medical research of osteogenic miRNAs on osteoporosis.

## Results

### miR-129-5p Was Associated With Bone Formation Reduction

**FIGURE 1:**
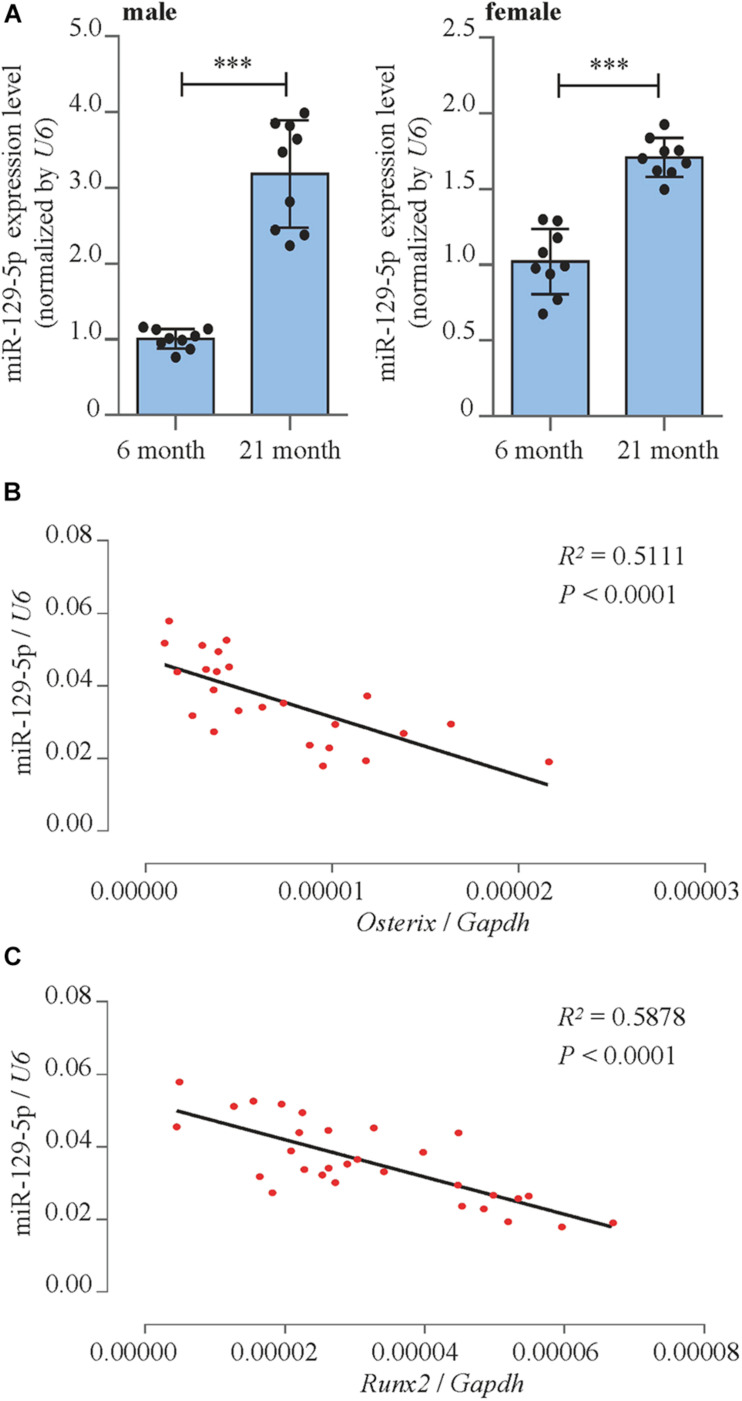
miR-129-5p was associated with bone formation reduction. **(A)** Expression levels of miR-129-5p in BMSCs of 6- and 21-month male (left) and female (right) C57BL/6 mice, as detected by reverse transcriptase-polymerase chain reaction (RT-PCR; mean ± SD, ^∗∗∗^*P* < 0.001). **(B,C)** Correlation analysis between miR-129-5p level and *Oxterix* or *Runx2* mRNA levels in femur tissues from C57BL/6 mice, respectively, as detected by RT-PCR.

We detected miR-129-5p expression level in bone marrow mesenchymal stem cells (BMSCs) of both male and female aging mice femur tissue. Reverse transcriptase-polymerase chain reaction (RT-PCR) results showed that in 21-month-old male and female mice, the expression level of miR-129-5p was enhanced by 218.3% (*P* < 0.001) and 70.7% (*P* < 0.001), respectively, compared to 6-month-old control mice (Figure 1A). These results implied that during the aging process, the expression of miR-129-5p is up-regulated in bone tissue and osteogenic cells.

To further prove the correlation between miR-129-5p and osteogenesis, correlation analysis was performed. The results showed that miR-129-5p expression was negatively correlated with the expression of osteogenic transcript factor *Osterix* and *Runx2* (runt-related transcription factor 2) in the femur tissue of different ages of C57BL/6 mice (Figures 1B,C). These results suggested that the expression level of miR-129-5p was negatively correlated with osteogenesis.

### miR-129-5p Inhibited Osteoblast Differentiation and Bone Formation

The functions of miR-129-5p on osteoblast differentiation and bone formation were investigated. miR-129-5p mimic and inhibitor were synthesized to manipulate miR-129-5p expression in MC3T3-E1 cells and calvaria of C57BL/6 mice. Mimic-NC and inhibitor-NC were used as control.

**FIGURE 2:**
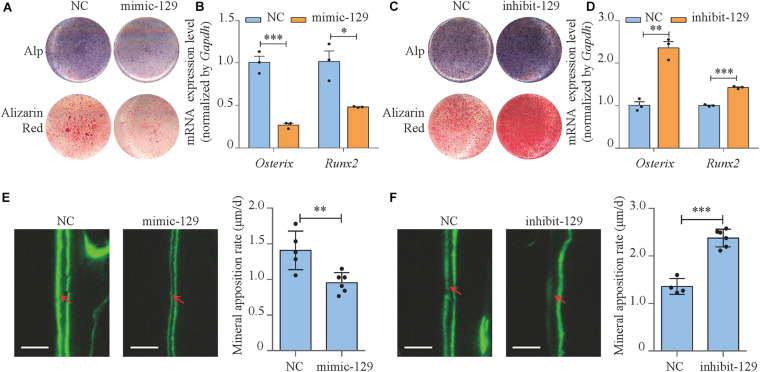
miR-129-5p inhibited osteoblast differentiation and bone formation. **(A)** Alp and alizarin red staining of MC3T3-E1 cells treated with mimic-129-5p (compared to mimic-NC), as detected by Alp staining and alizarin red staining. Alp, results of Alp staining; alizarin red, results of alizarin red staining; NC, mimic-NC; mimic-129, mimic-129-5p. **(B)** *Osterix* and *Runx2* expression levels of MC3T3-E1 cells treated with mimic-129-5p, as detected by reverse transcriptase-polymerase chain reaction (RT-PCR; mean ± SD, ^∗^*P* < 0.05, ^∗∗∗^*P* < 0.001). **(C)** Alp and alizarin red staining of MC3T3-E1 cells treated with inhibitor-129-5p (compared to inhibitor-NC), as detected by Alp staining and alizarin red staining. NC: inhibitor-NC. inhibit-129: inhibitor-129-5p. **(D)** *Osterix* and *Runx2* expression levels of MC3T3-E1 cells treated with inhibitor-129-5p, as detected by RT-PCR (mean ± SD, ^∗∗^*P* < 0.01, ^∗∗∗^*P* < 0.001). **(E)** Calvarial bone mineral apposition rate of C57BL/6 mice treated with mimic-129-5p (mean ± SD, ^∗∗^*P* < 0.01). Scale bar: 10 μm. **(F)** Calvarial bone mineral apposition rate of C57BL/6 mice treated with inhibitor-129-5p (mean ± SD, ^∗∗∗^*P* < 0.001). Scale bar: 10 μm.

In MC3T3-E1 cells, the expression level of miR-129-5p was increased by 123.7% (*P* < 0.001) compared to negative control after mimic-129-5p transfection (Supplementary Figure S1A) and was decreased by 37.8% (*P* < 0.001) when inhibitor-129-5p was transfected (Supplementary Figure S1B). RT-PCR results revealed that the expression level of osteogenic transcript factor *Osterix* and *Runx2* was decreased by 73% (*P* < 0.001) and 51.8% (*P* < 0.05), respectively when cells were exposed to mimic-129-5p (Figure 2B). Moreover, the alkaline phosphatase (ALP)-positive blue–violet complexes and alizarin red-stained mineralized nodules were both significantly decreased (Figure 2A and Supplementary Figure S2A). As for inhibitor-129-5p transfection, expression levels of *Osterix* and *Runx2* were enhanced by 135.5% (*P* < 0.01) and 42.4% (*P* < 0.001), respectively (Figure 2D). ALP-positive blue–violet complexes and alizarin red-stained mineralized nodules were also significantly enhanced (Figure 2C and Supplementary Figure S2B). The results confirmed that miR-129-5p inhibited osteoblast differentiation.

Then miR-129-5p mimic and inhibitor were implemented in calvaria of C57BL/6 mice by transfection reagent (Entranster^TM^ *In vivo* Transfection Reagent) to determine the function of miR-129-5p *in vivo*. The expression levels of miR-129-5p in mice calvarias were significantly enhanced by mimic-129-5p (Supplementary Figure S1C, *P* < 0.001) and decreased by inhibitor-129-5p (Supplementary Figure S1D, *P* < 0.001) 3 days after the injection. Mineral apposition rate (MAR, an assessment of bone formation) of calvarial bone in mimic-129-5p transfected mice was decreased by 32.2% (Figure 2E, *P* < 0.01), whereas calvarial MAR in mice treated by inhibitor-129-5p was enhanced by 75.1% (Figure 2F, *P* < 0.001). The *in vitro* and *in vivo* results proved that miR-129-5p would inhibit both osteoblast differentiation and bone formation.

### miR-129-5p Inhibited Downstream Transcript Factors of Wnt/β-Catenin Pathway

**FIGURE 3:**
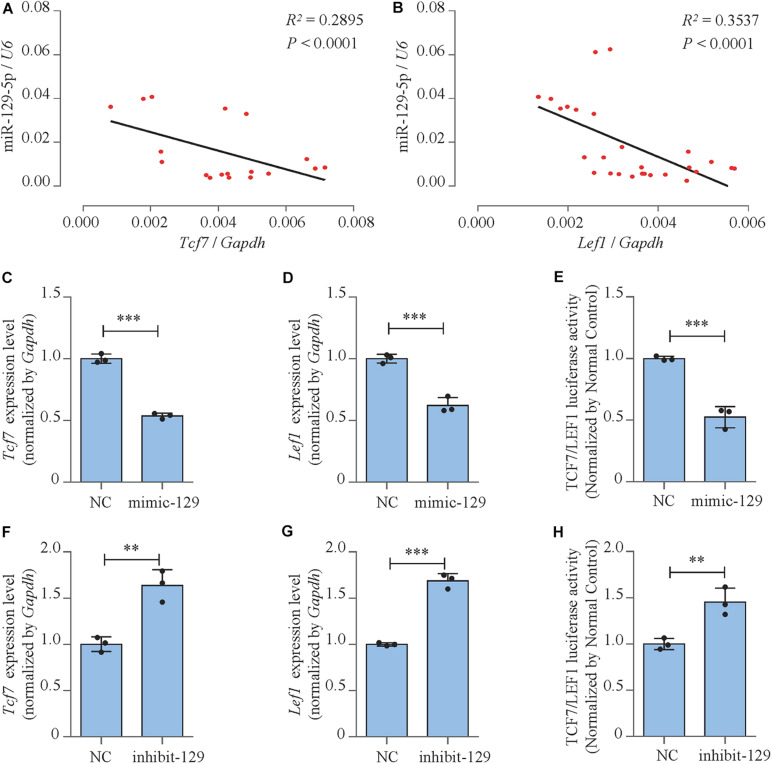
miR-129-5p inhibited downstream transcript factors of Wnt/β-catenin pathway. **(A,B)** Correlation analysis between miR-129-5p and *Tcf7* or *Lef1* mRNA levels in femur tissues from C57BL/6 mice, respectively, as detected by reverse transcriptase-polymerase chain reaction (RT-PCR). **(C,D)** *Tcf7* and *Lef1* expression levels of MC3T3-E1 cells treated with mimic-129-5p, as detected by RT-PCR (mean ± SD, ^∗∗∗^*P* < 0.001). NC, mimic-NC; mimic-129, mimic-129-5p. **(E)** TCF7/LEF1 activities of MC3T3-E1 cells treated with mimic-129-5p, as detected by luciferase reporter assay (mean ± SD, ^∗∗∗^*P* < 0.001). **(F,G)** *Tcf7* and *Lef1* expression levels of MC3T3-E1 cells treated with inhibitor-129-5p, as detected by RT-PCR (mean ± SD, ^∗∗^*P* < 0.01, ^∗∗∗^*P* < 0.001). NC, inhibitor-NC; inhibit-129, inhibitor-129-5p. **(H)** TCF7/LEF1 activities of MC3T3-E1 cells treated with inhibitor-129-5p, as detected by luciferase reporter assay (mean ± SD, ^∗∗^*P* < 0.01).

We moved forward to investigate the mechanism of the inhibitory effect of miR-129-5p on osteoblast differentiation. The correlation between miR-129-5p expression and essential osteogenic transcript factors in the femur tissue of different ages of C57BL/6 mice was investigated. The expression of transcript factor *Hes1*, *Smad2*, and *Hif1a* showed no significant correlation with miR-129-5p (Supplementary Figure S3). While *Tcf7* and *Lef1*, downstream transcript factors of Wnt/β-catenin pathway, showed negative correlation with miR-129-5p (Figures 3A,B and Supplementary Figure S4). Wnt/β-catenin pathway is one of the most essential pathways that regulate osteoblast differentiation and bone formation through its downstream transcript factors TCF7 and LEF1 (Maria et al., 2007; Hu et al., 2018). These results implied that miR-129-5p might inhibit downstream transcript factors of Wnt/β-catenin pathway.

The expression levels and activities of TCF7 and LEF1 in miR-129-5p mimic- or inhibitor-treated MC3T3-E1 cells were further determined. Results demonstrated that mRNA expression levels of *Tcf7* and *Lef1* were both significantly inhibited by the overexpression of miR-129-5p (Figures 3C,D; *P* < 0.001) and elevated by the knockdown of miR-129-5p (Figures 3F,G; *P* < 0.01, *P* < 0.001). Moreover, the negative influence of miR-129-5p on TCF7/LEF1 was confirmed by luciferase reporter assay. The luciferase reporter plasmid containing TCF7/LEF1 binding site was constructed and co-transfected into MC3T3-E1 cells along with miR-129-5p mimic or inhibitor. Results showed that after mimic or inhibitor-129-5p exposures, TCF7/LEF1 activities were evidently reduced (approximately 47.4%, Figure 3E, *P* < 0.001) or enhanced (45.4%, Figure 3H, *P* < 0.01). These data demonstrated that miR-129-5p had inhibitory effect on both the expressions and the activities of the downstream transcript factors of Wnt/β-catenin pathway, which suggested that the regulation of miR-129-5p on osteoblast differentiation was probably through the inhibition of these transcription factors.

### miR-129-5p Inhibited Osteoblast Differentiation and Wnt/β-Catenin Downstream Transcript Factors *via* Targeting *Tcf4*

**FIGURE 4:**
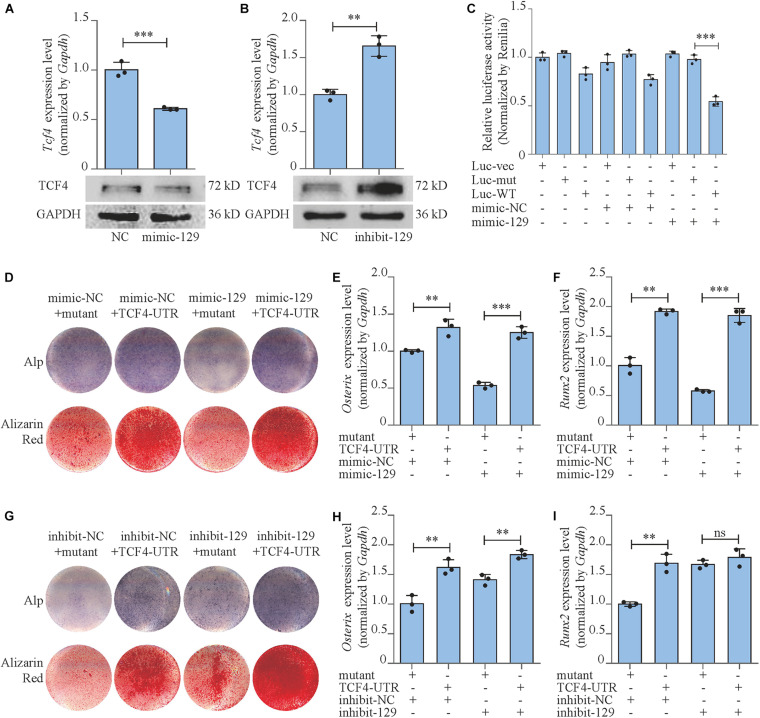
miR-129-5p inhibited osteoblast differentiation via targeting *Tcf4.* **(A,B)** TCF4 expression levels of MC3T3-E1 cells treated with mimic or inhibitor-129-5p, as detected by reverse transcriptase-polymerase chain reaction (RT-PCR) and Western blot (mean ± SD, ^∗∗∗^*P* < 0.001). NC, mimic-NC or inhibitor-NC; mimic-129, mimic-129-5p; inhibit-129, inhibitor-129-5p. **(C)** Binding effect of miR-129-5p and *Tcf4–*3′ UTR, as detected by luciferase reporter assay (mean ± SD, ^∗∗∗^*P* < 0.001). Luc-vec: empty luciferase reporter plasmid. Luc-mut: luciferase reporter plasmid containing mutant *Tcf4–*3′ UTR. Luc-WT: luciferase reporter plasmid containing wild-type *Tcf4–*3′ UTR. **(D)** Alp and alizarin red staining of MC3T3-E1 cells treated with *Tcf4–*3′ UTR plasmid and mimic-129-5p. Alp, results of Alp staining; alizarin red, results of alizarin red staining. Mutant: expression plasmid containing mutant miR-129-5p binding site sequence of *Tcf4–*3′ UTR. TCF4-UTR: expression plasmid containing wild-type miR-129-5p binding site sequence of *Tcf4–*3′ UTR. **(E,F)** *Osterix* or *Runx2* expression levels of MC3T3-E1 cells treated with *Tcf4–*3′ UTR plasmid and mimic-129-5p, as detected by RT-PCR (mean ± SD, ^∗∗^*P* < 0.01, ^∗∗∗^*P* < 0.001). **(G)** Alp and alizarin red staining of MC3T3-E1 cells treated with *Tcf4–*3′ UTR plasmid and inhibitor-129-5p. **(H,I)** *Osterix* or *Runx2* expression levels of MC3T3-E1 cells treated with *Tcf4–*3′ UTR plasmid and inhibitor-129-5p, as detected by RT-PCR (mean ± SD, ^∗∗^*P* < 0.01).

We have forecasted that miR-129-5p targeted *Tcf4* (Table 1), and TCF4 has been reported as an important conducting transcriptional factor in Wnt/β-catenin pathway (Reinhold and Naski, 2007). In this study, we for the first time determined the regulatory effect of miR-129-5p on TCF4. We discovered that mimic-129-5p significantly down-regulated TCF4 mRNA and protein expressions (Figure 4A), which were up-regulated by inhibitor-129-5p (Figure 4B). The binding effect of miR-129-5p to *Tcf4* -3′ UTR was also investigated by luciferase reporter assay. The luciferase reporter plasmids that either have a wild-type *Tcf4*–3′ UTR (Luc-WT) or a *Tcf4*–3′ UTR containing mutant sequences (Luc-mut) of the miR-129-5p binding site were constructed and transfected into MC3T3-E1 cells along with mimic-129-5p or inhibitor-129-5p. Results revealed that luciferase activities were significantly decreased by mimic-129-5p in cells transfected by Luc-WT (Figure 4C) and enhanced by inhibitor-129-5p (Supplementary Figure S5).

**TABLE 1:** Bonding sequence of miR-129-5p to *Tcf4* -3′UTR.

Sequences	Folding Energy (-Lcal/mol)
GTAA—-ACA—-AAGCAAAAAA | : | | | | | | | | | | |	−6.10
CGTTCGGGTCTGGCGTTTTTC	

To further confirm the role of TCF4 as a mediator between miR-129-5p and osteoblast differentiation, we constructed *Tcf4*-UTR plasmid that contained *Tcf4–*3′ UTR embodied miR-129-5p binding sequence. The control plasmid was also established that contained the mutant binding sequence (mutant) of miR-129-5p. *Tcf4*-UTR and mutant plasmid were transfected to MC3T3-E1 cells along with mimic-129-5p or inhibitor-129-5p. For MC3T3-E1 cells transfected with mimic-NC, osteoblast differentiation marker *Osterix* was up-regulated by 31.9% (*P* < 0.01) after treatment with *Tcf4–*3′ UTR, as compared to cells treated with mutant *Tcf4–*3′ UTR. In high-expression miR-129-5p cells induced by mimic-129-5p, *Osterix* was up-regulated by a more drastic level of 132.8% (*P* < 0.001) after *Tcf4–*3′ UTR treatment compared to mutant *Tcf4–*3′ UTR (Figure 4E). For *Runx2*, the similar patterns were observed as *Osterix*: *Tcf4–*3′ UTR enhanced its expression by 91.9% (*P* < 0.001) in cells transfected with mimic-NC, which was enhanced by a higher degree of 220% (*P* < 0.001) in mimic-129-5p-treated MC3T3-E1 cells (Figure 4F). Alp activities and mineralized nodules were also enhanced by *Tcf4–*3′ UTR, which were more significant in mimic-129-5p–treated cells (Figure 4D and Supplementary Figures S6A,B). On the other hand, in low miR-129-5p cells induced by inhibitor-129-5p, the expression levels of *Osterix* and *Runx2*, along with Alp activities and mineralized nodules were only slightly enhanced by *Tcf4–*3′ UTR as compared to cells with normal miR-129-5p level (Figures 4G–I and Supplementary Figures S6C,D). These results demonstrated that *Tcf4–*3′ UTR would alleviate the inhibitory effect of miR-129-5p on osteoblast differentiation and proved that miR-129-5p inhibited osteoblast differentiation via targeting *Tcf4*.

**FIGURE 5:**
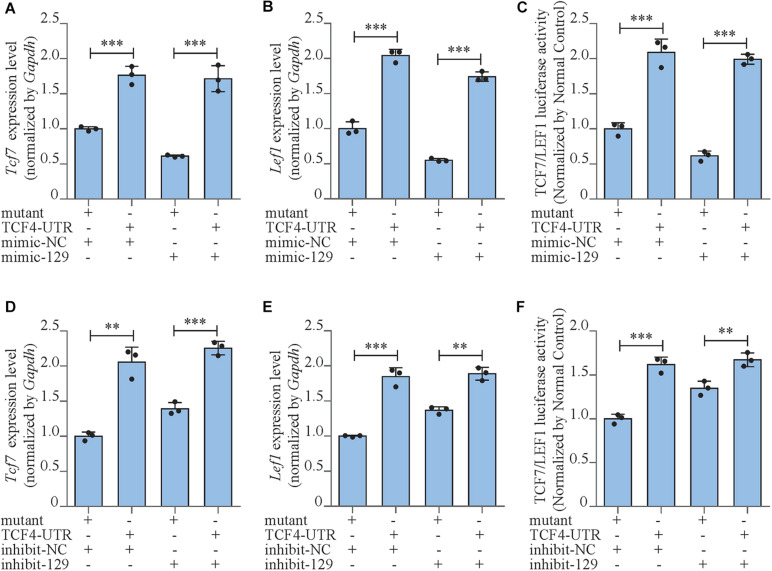
miR-129-5p inhibited Wnt/β-catenin downstream transcript factors via targeting *Tcf4.* **(A,B)** *Tcf7* and *Lef1* expression levels of MC3T3-E1 cells treated with *Tcf4–*3′ UTR plasmid and mimic-129-5p, as detected by reverse transcriptase-polymerase chain reaction (RT-PCR; mean ± SD, ^∗∗∗^*P* < 0.001). Mutant/TCF4-UTR: expression plasmid containing mutant/wild-type miR-129-5p binding site sequence of *Tcf4–*3′ UTR. **(C)** TCF7/LEF1 activities of MC3T3-E1 cells treated with *Tcf4–*3′ UTR plasmid and mimic-129-5p, as detected by luciferase reporter assay (mean ± SD, ^∗∗∗^*P* < 0.001). **(D,E)** *Tcf7* and *Lef1* expression levels of MC3T3-E1 cells treated with *Tcf4–*3′ UTR plasmid and inhibitor-129-5p, as detected by RT-PCR (mean ± SD, ^∗∗^*P* < 0.01, ^∗∗∗^*P* < 0.001). **(F)** TCF7/LEF1 activities of MC3T3-E1 cells treated with *Tcf4–*3′ UTR plasmid and inhibitor-129-5p, as detected by luciferase reporter assay (mean ± SD, ^∗∗^*P* < 0.01, ^∗∗∗^*P* < 0.001).

Moreover, we also investigated the expressions and the activities of the downstream transcript factors of Wnt/β-catenin pathway in *Tcf4*-UTR transfected MC3T3-E1 cells. The treatment of *Tcf4*-UTR up-regulated mRNA expression levels of *Tcf7* and *Lef1* by 76.4% (*P* < 0.001) and 104.3% (*P* < 0.001), respectively in mimic-NC-treated cells. However, in high-expression miR-129-5p cells, *Tcf4*-UTR up-regulated *Tcf7* and *Lef1* level up to 180% (*P* < 0.001) and 215.3% (*P* < 0.001), respectively (Figures 5A,B). The activity of TCF7/LEF1 was enhanced by 108.9% (*P* < 0.001) through *Tcf4*-UTR treatment in cells with normal miR-129-5p level and reached 223.8% (*P* < 0.001) in high miR-129-5p cells (Figure 5C). Meanwhile, after inhibitor-129-5p treatment, *Tcf4*-UTR enhanced both expressions and activities of TCF7 and LEF1 by a minor extent than that in cells with normal miR-129-5p level (Figures 5D–F). All these results indicated that miR-129-5p inhibited osteoblast differentiation and downstream transcript factors of Wnt/β-catenin pathway through targeting *Tcf4*.

### Rescue Effect of miR-129-5p Inhibitor on Postmenopausal Osteoporosis

**FIGURE 6:**
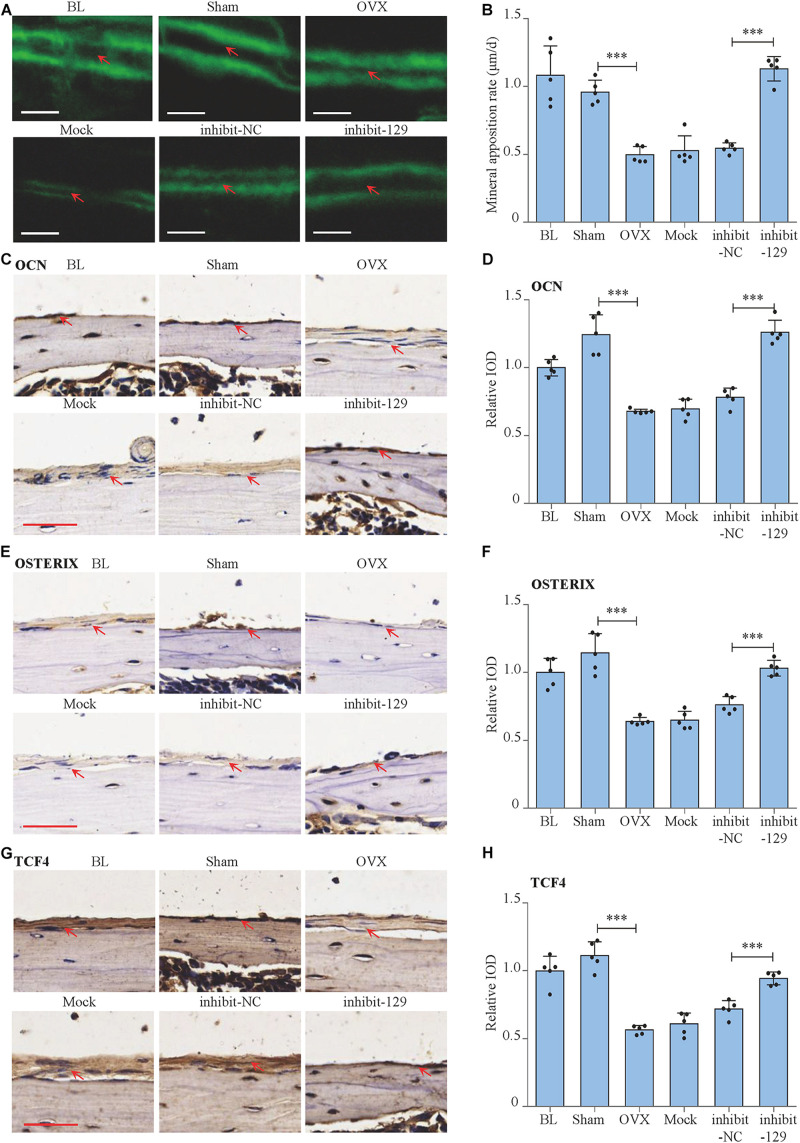
Rescue effect of miR-129-5p inhibitor on postmenopausal osteoporosis. **(A)** Representative images showing calvarial mineral apposition rate of C57BL/6 mice after OVX and inhibitor-129-5p treatment. Scale bar: 10 μm. BL (baseline): sacrifice before RNA treatment. Sham: sham OVX operation group. OVX: OVX group. Mock: transfection reagent control group. inhibit-NC: inhibitor-NC–treated group. inhibit-129: inhibitor-129-5p–treated group. **(B)** Calvarial mineral apposition rates of C57BL/6 mice after OVX and inhibitor-129-5p treatment (mean ± SD, ^∗∗∗^*P* < 0.001). **(C)** Expression of OCN in calvarial tissues of C57BL/6 mice after OVX and inhibitor-129-5p treatment, as detected by immunohistochemical staining. Scale bar: 50 μm. **(D)** Quantification of relative integrated optical density (IOD) values of OCN immunostaining using Image-Pro Plus 6.0 software (mean ± SD, ^∗∗∗^*P* < 0.001). **(E)** Expression of OXTERIX in calvarial tissues of C57BL/6 mice after OVX and inhibitor-129-5p treatment, as detected by immunohistochemical staining. Scale bar: 50 μm. **(F)** Quantification of relative IOD values of OXTERIX immunostaining using Image-Pro Plus 6.0 software (mean ± SD, ^∗∗∗^*P* < 0.001). **(G)** Expression of RUNX2 in calvarial tissues of C57BL/6 mice after OVX and inhibitor-129-5p treatment, as detected by immunohistochemical staining. Scale bar: 50 μm. **(H)** Quantification of relative IOD values of RUNX2 immunostaining using Image-Pro Plus 6.0 software (mean ± SD, ^∗∗∗^*P* < 0.001).

**FIGURE 7:**
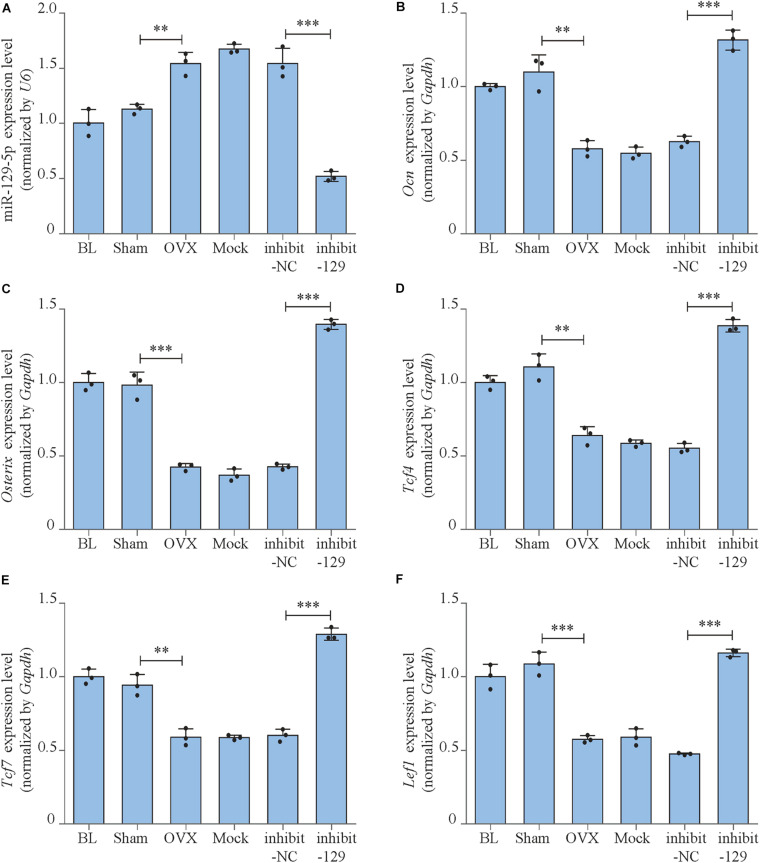
miR-129-5p and osteogenic transcript factor expression levels of OVX mice after inhibitor-129-5p treatment. **(A)** miR-129-5p level in calvarial tissue of C57BL/6 mice after OVX and inhibitor-129-5p treatment, as detected by reverse transcriptase-polymerase chain reaction (RT-PCR; mean ± SD, ^∗∗^*P* < 0.01, ^∗∗∗^*P* < 0.001). BL (baseline): sacrifice before RNA treatment. Sham: sham OVX operation group. OVX: OVX group. Mock: transfection reagent control group. inhibit-NC: inhibitor-NC–treated group. inhibit-129: inhibitor-129-5p–treated group. **(B–F)** *Ocn*, *Osterix*, *Tcf4*, *Tcf7*, and *Lef1* expression levels in calvarial tissue of C57BL/6 mice after OVX and inhibitor-129-5p treatment, as detected by RT-PCR (mean ± SD, ^∗∗^*P* < 0.01, ^∗∗∗^*P* < 0.001).

We moved forward to investigate the rescue effect of miR-129-5p inhibitor on osteoporosis. Postmenopausal osteoporosis mouse model was constructed by ovariectomization. The transfection of inhibitor-129-5p was implemented into calvarias of OVX mice as previously described (Yin et al., 2019). Expression levels of miR-129-5p in calvarias were significantly enhanced by OVX surgery and were significantly down-regulated upon transfection (Figure 7A). Mineral apposition rate in the OVX mice was decreased by 48.2% (*P* < 0.001) and increased by 107.3% (*P* < 0.001) after treatment with inhibitor-129-5p (Figures 6A,B). Moreover, the expression levels of osteogenic factors OCN and OSTREIX, along with miR-129-5p target gene TCF4, were all down-regulated after OVX surgery, which were rescued by inhibitor-129-5p treatment. mRNA expression level of transcript factors *Tcf7* and *Lef1* also exhibited similar tendency (Figures 6C–H, 7B–F). The results proved the rescue effect of inhibitor-129-5p on postmenopausal osteoporosis, and further implied that miR-129-5p might serve as a potential therapeutics target for osteoporosis.

### Bioengineered Recombinant miR-129-5p Inhibitor Enhanced Osteoblast Differentiation and Rescued Postmenopausal Osteoporosis

**FIGURE 8:**
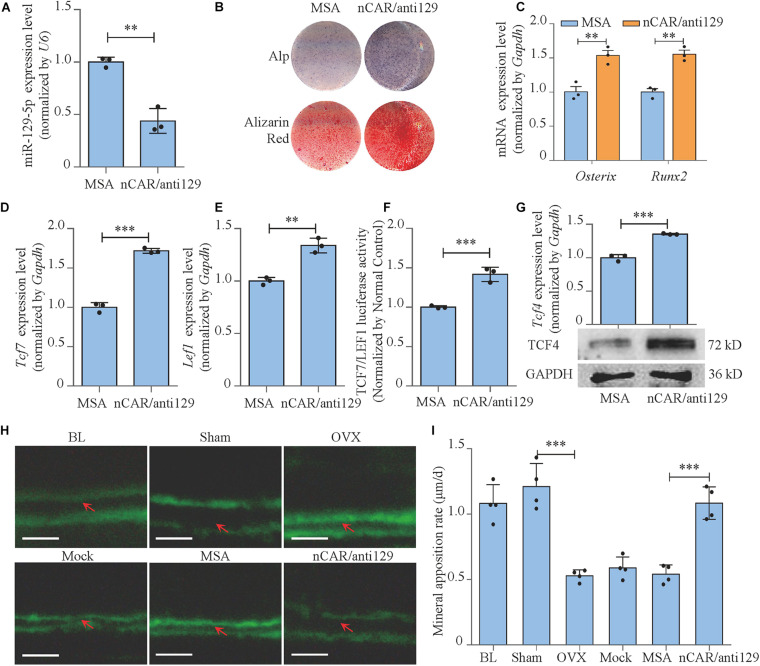
Bioengineered recombinant miR-129-5p inhibitor enhanced osteoblast differentiation and rescued postmenopausal osteoporosis. **(A)** miR-129-5p expression levels of MC3T3-E1 cells treated with recombinant miR-129-5p inhibitor, as detected by reverse transcriptase-polymerase chain reaction (RT-PCR; mean ± SD, ^∗∗^*P* < 0.01). MSA: tRNA^Met^ fused Sephadex aptamer. nCAR/anti129: novel recombinant miR-129-5p inhibitor. **(B)** Alp and alizarin red staining of MC3T3-E1 cells treated with recombinant miR-129-5p inhibitor, as detected by Alp staining and alizarin red staining. Alp: results of Alp staining. alizarin red: results of alizarin red staining. **(C)** *Osterix* and *Runx2* expression levels of MC3T3-E1 cells treated with recombinant miR-129-5p inhibitor, as detected by RT-PCR (mean ± SD, ^∗∗^*P* < 0.01). **(D,E)** *Tcf7* and *Lef1* expression levels of MC3T3-E1 cells treated with recombinant miR-129-5p inhibitor, as detected by RT-PCR (mean ± SD, ^∗∗^*P* < 0.01, ^∗∗∗^*P* < 0.001). **(F)** TCF7/LEF1 activities of MC3T3-E1 cells treated with recombinant miR-129-5p inhibitor, as detected by luciferase reporter assay (mean ± SD, ^∗∗∗^*P* < 0.001). **(G)** TCF4 expression levels of MC3T3-E1 cells treated with recombinant miR-129-5p inhibitor, as detected by RT-PCR and Western blot (mean ± SD, ^∗∗∗^*P* < 0.001). **(H)** Representative images showing calvarial mineral apposition rate of C57BL/6 mice after OVX and recombinant miR-129-5p inhibitor treatment. Scale bar: 10 μm. BL (baseline): sacrifice before RNA treatment. Sham: sham OVX operation group. OVX: OVX group. Mock: transfection reagent control group. MSA: empty recombinant tRNA treated group. nCAR/anti129: novel recombinant miR-129-5p–inhibitor treated group. **(I)** Calvarial mineral apposition rates of C57BL/6 mice after OVX and recombinant miR-129-5p inhibitor treatment (mean ± SD, ^∗∗∗^*P* < 0.001).

To further achieve the translational medical application of miR-129-5p with higher efficiency and lower cost, bioengineered novel recombinant miR-129-5p inhibitor was manufactured through *E. coli* system (Supplementary Figure S7), and its function was further determined. The recombinant miR-129-5p inhibitor efficiently decreased miR-129-5p level in MC3T3-E1 cells (approximately 56%, Figure 8A, *P* < 0.01). The Alp activities and mineralized nodules, along with *Osterix* and *Runx2* expression level, were all significantly enhanced, indicating that the recombinant miR-129-5p inhibitor promoted osteoblast differentiation (Figures 8B,C and Supplementary Figure S8). TCF and LEF1 presented a significant increment in both mRNA expressions and activities (Figures 8D–F). Expression of miR-129-5p target gene TCF4 was also significantly increased (Figure 8G), demonstrating that recombinant miR-129-5p inhibitor enhanced TCF4 level and downstream transcript factors of Wnt/β-catenin pathway. Moreover, recombinant miR-129-5p inhibitor was also implemented in calvarias of OVX mice. The mineral apposition rate was decreased by 56.3% (*P* < 0.001) in the OVX mice, but was increased by 100.2% (*P* < 0.001) upon recombinant miR-129-5p inhibitor treatment (Figures 8H–I). The *in vitro* and *in vivo* results have proved the prospect that recombinant miR-129-5p inhibitor may be used as a potential therapeutic drug for osteoporosis.

## Discussion

Osteoporosis has become an emerging threat to elderly population because of its high incidence. The patients would suffer from bone mass reduction, bone microstructure deterioration, hunchback, muscle spasms, myasthenia, pain, and increased risk of fracture. Many factors contribute to osteoporosis, including genetic diseases, hormonal disruption, malnutrition, drug side effects, disuse, etc. (Georgiou et al., 2012). Among all reasons, one of the most essential causes of osteoporosis is the deterioration of osteoblast differentiation, which decreased bone formation and further led to osteoporosis.

The differentiation of osteoblast is a long-term physiological process that could be impacted by many genetic and epigenetic factors. miRNAs are a sort of non-coding RNA with 18 to 25 nucleotides in length. miRNAs are widely expressed in eukaryotes and have been known as important regulators for multiple physiological and pathological processes (Bartel, 2009; Rigoutsos and Furnari, 2010; Chen et al., 2017). miRNAs played an important role in regulating osteoblast differentiation and bone formation. Wang et al. revealed that miR-214 inhibited bone formation *via* targeting *Atf4* (Wang et al., 2013). Li et al. (2015) have reported that miR-188 inhibited osteogenic differentiation of BMSCs and consequently promoted BMSC adipogenic differentiation, which further mediated aging-related osteoporosis. Zuo et al. (2015) discovered miR-103a, which was sensitive to mechanical stimulation and mediated disuse osteoporosis by targeting *Runx2*. Wang et al. (2018, 2020) recently discovered that miR-139-3p inhibited osteoblast function and bone formation through *Elk1*. These studies demonstrated the important role of miRNAs on bone formation and implied that translational medical research of osteogenic miRNAs would be helpful to discover potential treatment for osteoporosis.

miR-129-5p is a miRNA that has been reported as a regulator of cancer development and Alzheimer disease (Li G. et al., 2019; Zeng et al., 2019). Shi et al. found hsa-miR-129-5p inhibited osteogenic differentiation of adipose-derived stem cells (Shi et al., 2020), while its function on bone formation is still unexplored. To further reveal the correlation between miR-129-5p and osteoporosis, in this study, we have stimulated aging-related osteoporosis in male aging mice model and menopause osteoporosis in female aging menopause mice model. The expression of miR-129-5p in BMSC demonstrated that miR-129-5p expression was enhanced in both osteoporosis models. Moreover, correlation analysis also proved that miR-129-5p negatively correlated with osteogenic marker gene in different ages of mice (Figure 1). We further proved the inhibitory effect of miR-129-5p on osteoblast differentiation and bone formation (Figure 2). The results proved that miR-129-5p is an important regulatory factor to osteoblast differentiation and bone formation.

MicroRNA inhibited the translation of target gene by binding to its 3′ UTR region (Lai, 2002). In Shi et al.’s (2020) study, hsa-miR-129-5p inhibited Wnt/β-catenin pathway via targeting *Grm5*. In Li et al. (2013); Zhang et al. (2017), and Cao et al. (2018), hsa-miR-129-5p inhibited Wnt/β-catenin pathway via targeting *Apc*, *Sox4*, and *Wnt5a*, respectively. Wnt/β-catenin pathway is one of the most essential pathways regulating bone formation, which activates multiple osteogenic factors by TCF7 and LEF1 (Maria et al., 2007; Georgiou et al., 2012). In our study, we have first proved that miR-129-5p inhibited the expression and activity of downstream transcription factors of Wnt/β-catenin pathway TCF7 and LEF1 (Figure 3). However, *Tcf7* and *Lef1* were not target genes of miR-129-5p (predicted by miRDB, http://www.mirdb.org/, Chen and Wang, 2020), so we turned our attention to the regulator of *Tcf7* and *Lef1*. Because multiple TCFs within wnt signaling pathway played essential roles on regulating *Tcf7* and *Lef1*. Therefore, we used miRDB to screen the targeting effect of miR-129-5p on TCFs and found the 3′UTR of Tcf4 had 6 binding sites of miR-129-5p. TCF4 has been reported as an important regulatory factor of wnt/β-catenin pathway, and therefore promoted osteogenic differentiation (Reinhold and Naski, 2007). On this basis, we confirmed the targeting effect of miR-129-5p on *Tcf4*. Moreover, the 3′UTR sequence of *Tcf4* was also proved to enhance both osteoblast differentiation and downstream transcription factors of wnt/β-catenin pathway (Figures 4, 5). All these results illustrated that miR-129-5p promoted osteoblast differentiation and bone formation through inhibiting downstream transcription factors of wnt/β-catenin pathway by targeting *Tcf4*. However, besides TCF4, other unknown mechanisms may also exist for the inhibition of osteoblast differentiation by miR-129-5p.

Based on the finding that miR-129-5p inhibited bone formation, we have further proved that the inhibitor of miR-129-5p ameliorated menopausal osteoporosis (Figures 6, 7). However, until now, most RNA-based therapeutic drugs were still manufactured by chemical synthesis, which resulted in very high cost on synthesis and the unclear biosafety. The novel RNA bioengineering system that can express functional miRNA and siRNA by *E. coli* was recently established (Ho et al., 2018; Li X. et al., 2019), which could manufacture recombinant RNA with extremely low cost (multi-mg RNA in 1L *E. coli*), along with high safety and high efficiency. Using this technique, the novel recombinant miR-129-5p inhibitor was manufactured, and its effects on osteoblast have been proved as good as chemical synthesized miR-129-5p inhibitor. Moreover, the novel recombinant miR-129-5p inhibitor also proved its high efficiency on rescuing menopausal osteoporosis at much lower dose (four times less) compared to chemical synthesized miR-129-5p inhibitor (Figure 8). These results have proved that the novel recombinant miR-129-5p inhibitor may serve as a promising drug for osteoporosis treatment.

Taken together, we have identified miR-129-5p that inhibited osteoblast differentiation and bone formation. miR-129-5p targeted *Tcf4* and thus inhibited downstream transcription factors of Wnt/β-catenin pathway. miR-129-5p inhibitor ameliorated menopausal osteoporosis, and the novel recombinant miR-129-5p inhibitor showed its high efficiency on promoting osteoblast differentiation and rescuing osteoporosis. The study illuminated a new mechanism regulating bone formation and also provided novel therapeutic strategies for the treatment of osteoporosis.

## Materials and Methods

### Cell Culture and Mouse Model

Murine preosteoblast MC3T3-E1 cell line was generously provided by Dr. Hong Zhou (The University of Sydney, Sydney, Australia). MC3T3-E1 cells were cultured in osteoblast culture medium containing α modified eagle medium (α-MEM, Gibco, 11900-024, Carlsbad, CA) supplemented with 10% fetal bovine serum (FBS; Biological Industries, 04-001-1A, Kibbutz Beit Haemek, Israel), 1% L-glutamine (Sigma, G8540, St. Louis, MO, United States), 1% penicillin (Amresco, 0242, Solon, OH, United States), and streptomycin (Amresco, 0382, Solon, OH, United States). Cell cultures were maintained at a humidified 37°C, 5% CO_2_ incubator (Thermo Fisher Scientific, Waltham, MA, United States). Murine mesenchymal stem cell line C3H10 T1/2 was purchased from Runde Biotechnology Co., Ltd. (Xi’an, China). C3H10 T1/2 cells were cultured in mesenchymal stem cell culture medium containing Dulbecco modified eagle medium (DMEM, Gibco, 12800-017) supplemented with 10% FBS, 1% L-glutamine, 1% penicillin, and streptomycin. Cell cultures were maintained at a humidified 37°C, 5% CO_2_ incubator.

Aging and ovariectomized (OVX) mice were adopted to construct the osteoporosis model. All mice were purchased from the Laboratory Animal Center of the Fourth Military Medical University (Xi’an, China). For aging mice model, 12 male and 12 female 6-month-old male C57BL/6 mice were maintained under standard animal housing conditions (12-h light, 12-h dark cycles and free access to food and water). Six male mice and six female mice which, were kept until 21 months old, were selected as aging group, whereas the other mice were used as control group. Mice were euthanized, and femurs were collected and processed for RNA-seq or BMSCs isolation for RT-PCR.

For OVX mouse model, sixty-six 2-month-old female C57BL/6 mice were maintained under standard animal housing conditions. The mice were ovariectomized or sham-operated at 3 months of age. Mice were euthanized 38 days after surgery (4 months of age), and the calvarias were collected to investigate the therapeutic effects of miR-129-5p inhibitor and recombinant miR-129-5p inhibitor. Euthanasia was performed using CO_2_. All animal experiments were performed in accordance with the recommendation of the Guiding Principles for the Care and Use of Laboratory Animals (the Institutional Experimental Animal Committee of Northwestern Polytechnical University, Xi’an, China), and all animal studies were reviewed and approved by the Institutional Experimental Animal Committee of Northwestern Polytechnical University, Xi’an, China. For all procedures involving animals, all efforts were made to reduce the number of the mice used and their suffering.

### Isolation of BMSCs

Mouse BMSCs were isolated to investigate the expression levels of miR-129-5p in male and female aging mice. Mouse femurs were immediately harvested, and attached soft tissues were removed. Bone marrow was washed and collected by phosphate-buffered saline (PBS) using a 25-gauge syringe needle. The collected PBS with bone marrow was centrifuged (1,200*g*, 8 min) and dissociated by culture medium using a 29-gauge syringe needle. The suspension was cultured in a 60-mm plate for 3 h (37°C, 5% CO_2_), and culture medium was changed. Cells were cultured for another 36 h with culture medium changed every 12 h. The cells were transferred into a new plate as the first-passage cells. Third-passage cells were used for RT-PCR detection.

### Real-Time PCR

Real-Time PCR was used to assess expression levels of miR-129-5p and selected mRNAs. Total RNA was extracted from mouse femur and calvarial tissues or culture cells using Trizol reagent (Invitrogen, 15596018). Mouse femur and calvaria were harvested and grinded with liquid nitrogen and then digested by Trizol reagent; 1 μg of total RNA was used for cDNA synthesis using one-step PrimeScript RT reagent kit (TaKaRa, RR037A, Dalian, China). Quantitative PCR amplification was performed using the Thermal Cycler C-1000 Touch system (BIO-RAD CFX Manager, Hercules, CA) and SYBR Premix Ex TaqII kit (TaKaRa, RR820A). For mRNA, *Gapdh* was used as internal control gene. For miR-129-5p, *U6* was used as internal control. The quantitative PCR reaction conditions included initial denaturation step at 95°C for 30 s, followed by 42 cycles at 95°C for 10 s, 60°C for 30 s, and 72°C for 5 s. Data were calculated using the comparative Ct method (2^–Δ^ ^Δ^ ^Ct^) and expressed as fold change compared to corresponding control. Primers (for sequences, see Table 2) were synthesized by Sangon Biotech Co., Ltd. (Shanghai, China).

**TABLE 2:** Primer Sequences for qRT-PCR.

Target gene	Sequences (5→′3′)
miR-129-5p-Forward	CTTTTTGCGGTCTGGGCTTGC
miR-129-5p-Reverse	AGTGCAGGGTCCGAGGTATT
miR-129-5p-RT	GTCGTATCCAGTGCAGGGTCCGAGGTATTCGCA CTGGATACGACGCAAGC
*Osterix*-Forward	CCCTTCCCTCACTCATTTCC
*Osterix*- Reverse	CAACCGCCTTGGGCTTAT
*Runx2*-Forward	CGCCCCTCCCTGAACTCT
*Runx2*-Reverse	TGCCTGCCTGGGATCTGTA
*Tcf4*-Forward	ATCGCAGACGCAAGAGGTTTCAG
*Tcf4*-Reverse	ACATACCGCTTCGCACATTCAGAG
*Tcf7*-Forward	CAGAATCCACAGATACAGCA
*Tcf7*-Reverse	CAGCCTTTGAAATCTTCATC
*Lef1*-Forward	GATCCCCTTCAAGGACGAAG
*Lef1*-Reverse	GGCTTGTCTGACCACCTCAT
*Ocn*-Forward	GAAGGCAACAGTCGATTCACC
*Ocn*-Reverse	GACTGTCTTGCCCCAAGTTCC
*Gapdh*-Forward	TGCACCACCAACTGCTTAG
*Gapdh*- Reverse	GGATGCAGGGATGATGTTC
*U6*-Forward	GTGCTCGCTTCGGCAGCACATAT
*U6*-Reverse	AAAATATGGAACGCTTCACGAA

### Transfection of the miR-129-5p Mimic and Inhibitor

miR-129-5p mimic and inhibitor (Genepharma, Shanghai, China) transfection was performed to manipulate miR-129-5p expression. For transfection *in vitro*, MC3T3-E1 or C3H10 T1/2 cells were seeded in a 24-well plate at 3.2 × 10^4^ cells per well and were transfected with miR-129-5p mimic or inhibitor by Lipofectamine^TM^ 2000 (Invitrogen, 11668-030) according to the manufacturer’s instructions (40 nM), using mimic-NC or inhibitor-NC as normal control, respectively. The overexpression or knockdown of miR-129-5p in cells was confirmed by RT-PCR 36 h after the transfection.

For *in vivo* transfection, 32 female C57BL/6 mice (4-month-old) were randomly divided into four groups (mimic-NC, mimic-129-5p, inhibitor-NC, inhibitor-129-5p). For each group, mice were injected subcutaneously over the calvarial surface with plasmids formulated with Entranster^TM^ *In Vivo* Transfection Reagent (Engreen, 18668-11-2, Beijing, China) at the dosage of 40 μL (including 4.8 μg RNA) according to the manufacturer’s instructions. All mice received the same standard diet during the experimental period. Three mice from each group were euthanized 3 days after treatment, and calvarias from mice were processed for RT-PCR (*n* = 3/group). All other mice were euthanized 21 days after treatment, and calvarias were processed for histomorphometric analyses (*n* = 5/group; Yin et al., 2019).

### Alkaline Phosphatase Staining and Alizarin Red Staining

For osteogenic differentiation treatment, MC3T3-E1 cells at confluence of 100% were induced by osteogenic medium with α-MEM, 10% FBS, 1% β-glycerophosphate (Sigma, G9422), 1% ascorbic acid (Sigma, A7631), 1% penicillin/streptomycin, and 1% L-glutamine. The cell cultures were maintained at 37°C with 5% CO_2_, and medium was replaced every 2 days. As for C3H10 T1/2 cells, osteogenic medium was made by DMEM, 20% FBS (ExCell Bio, FND500, Moorebank, Australia), 1% β-glycerophosphate, 1% ascorbic acid, 1% penicillin/streptomycin, 1% L-glutamine, and 1 mM dexamethasone (Sigma, D4902).

Alp staining and alizarin red staining were performed to determine osteoblast differentiation. ALP of osteoblasts was stained 3 days after having been induced by osteogenic medium. The staining was performed by 5-bromo-4-chloro-3-indolyl phosphate (BCIP)/ nitro blue tetrazolium (NBT) Alkaline Phosphatase Color Development Kit (Beyotime Biotechnology, C3206, Shanghai, China) according to the manufacturer’s instruction. Briefly, cells were washed with PBS (pH7.4) and fixed in 10% buffered formaldehyde. Formaldehyde was washed with PBS, and then cells were stained with BCIP/NBT solution. The staining was stopped by immersing into distilled water. After staining, the plates were dried and scanned with CanoScan 9000F Mark II scanner (Canon, Tokyo, Japan). For MC3T3-E1 cells, alizarin red staining was carried out after having been induced by osteogenic medium for 21 days. As for C3H10 T1/2 cells, alizarin red staining was carried out after 10 days’ treatment of osteogenic medium. The cells were washed with PBS and then stained with 0.5% alizarin red S (pH 4.0, Sigma, A5533) for 30 min. After immersion into tap water for 30 min, the plates were dried and scanned with CanoScan 9000F Mark II scanner and analyzed by Image-Pro Plus 6.0 software (National Institutes of Health, Bethesda, MD) to determine the ALP and alizarin red staining intensities.

### Western Blot

For detection of protein levels, Western blot analysis was performed as previously described (Yin et al., 2018). Protein samples from cultural cells were extracted. Cells were washed three times by cold PBS and then digested by cell lysis buffer (Beyotime, P0013, Haimen, China) with 1% protease inhibitor cocktail (Calbiochem, 539134, Darmstadt, Germany). Protein concentrations were analyzed by bicinchoninic acid protein assay kit (Thermo Fisher Scientific, 23225); 100 ng of proteins for each sample was subjected to sodium dodecyl sulfate–polyacrylamide gel electrophoresis (PAGE) using 5% stacking gel and 12% separating gel, 140 V, 30 min, and transferred (400 mA, 1 h) to nitrocellulose filter membranes (Pall, 66485, Port Washington, NY, United States). Membranes were blocked with 4% skimmed milk (BD Biosciences, 232100, Franklin Lakes, NJ, United States) for 1 h at room temperature and then incubated with primary antibodies at 4°C overnight with the following primary antibodies: TCF4 (Rabbit pAb, 1:1,000, Proteintech, 22337-1-AP, Rosemont, IL, United States) and GAPDH (Rabbit pAb, 1:1,000; Proteintech, 10494-1-AP). Blots were then incubated with horseradish peroxidase (HRP)–labeled secondary antibody (1:2,000; CWBIO, CW0103, Beijing, China) and visualized using chemiluminescence detection system (Thermo Fisher Scientific, NCI5080). Protein bands were scanned by a chemiluminescence imaging system (Tanon, 4600SF, Shanghai, China). GAPDH was adopted as internal control.

### Bone Histomorphometric Analyses

Bone histomorphometric analysis was performed to investigate the effect of miR-129-5p on bone formation. To measure mineral appositional rate, double-calcein labeling was performed by intraperitoneal injection with calcein green (20mg/kg body weight, Sigma, C0875) in the time sequence of 10 and 3 days before euthanasia for specimen collection. Collected calvarial samples were directly embedded in OCT (Leica, 14020108926, Wetzlar, Germany). Transverse cryosections (4 μm in thickness) were made by a freezing-microtome (Leica, CM1100), and slides were examined with a fluorescent microscope (NEXCOPE NIB900, Ningbo, China). Bone dynamic histomorphometric analyses for mineral apposition rate were performed using image analysis software (Image J, National Institutes of Health, Bethesda, MD, United States; Ushiku et al., 2010).

### Immunohistochemical Staining

To investigate the rescue effect of miR-129-5p inhibitor on osteogenic gene and TCF4 levels in mice calvarias, immunohistochemical staining analysis was performed as previously described (Yin et al., 2019). Mouse calvarias were dissected and fixed in 4% paraformaldehyde, decalcified in 17% ethylenediaminetetraacetic acid (Sigma, E9884) for 21 days, and embedded in paraffin (Huayong, Shanghai, China). Sections (5 μm in thickness) were dewaxed, immersed in the distilled water, blocked in 5% goat serum (CWBIO, CW0130) in PBS, and then incubated overnight at 4°C with primary antibodies against OCN (Rabbit pAb, 1:200, Santa Cruz Biotech, sc-365797, Dallas, TX, United States), OSTERIX (Rabbit pAb, 1:50; Proteintech, 12593-1-AP), and TCF4 (Rabbit pAb, 1:100, Proteintech, 22337-1-AP), respectively. Following three washes in PBS, the sections were labeled with HRP-labeled secondary antibody 1.5 h at room temperature and developed for color reaction using diaminobenzidine (CWBIO, CW2068) and hematoxylin counterstain. Slides were scanned by Aperio AT2 Digital Pathology Scanner (Leica), and protein immunostaining intensities on top surface of the calvarias were analyzed by Image-Pro Plus 6.0 software.

### Luciferase Reporter Assay

To analyze the function of miR-129-5p in regulating TCF7/LEF1 activity in MC3T3-E1 cells, TCF7/LEF1 luciferase reporter plasmid was constructed by inserting eight repeats of TCF7/LEF1 binding motif sequence (AGATCAAAGG) to the promoter region of nanoluc luciferase gene sequence in PNL1.1 plasmid (N1351, Promega, Fitchburg, WI). MC3T3-E1 cells were seeded in a 6-well plate at 1 × 10^5^ cells per well, and TCF7/LEF1 luciferase reporter plasmid was transfected by Engreen Entranster^TM^ H4000 Reagent according to the manufacturer’s instructions. Six hours after transfection, the medium was replaced by antibiotic-free culture medium. After 3-h culture, the cells were transfected with miR-129-5p mimic or inhibitor (40 nM) with its relative mimic-NC or inhibitor-NC as negative control. A luciferase reporter assay was performed 72 h after the transfection using the Nano-Glo^®^ Luciferase Assay System (Promega, N1120, Fitchburg, WI, United States) according to the manufacturer’s instruction. Briefly, 100 μL cell culture medium was collected into a microplate, and then 100 μL diluted Nano-Glo^®^ Luciferase Assay Substrate was added. Nanoluc luciferase luminescent signals were quantified by a microplate reader (Synergy, United States) at 460 nm, and each value from the nanoluc luciferase constructs was normalized by a normal control.

To detect interaction between miR-129-5p and *Tcf4*, wild-type binding sequence of miR-129-5p in *Tcf4*–3′ UTR (Luc-WT, with binding site sequence “GCAAAAAA”) and its relative mutant binding sequence (Luc-mut, with binding site sequence “TAGGGGGG”) were designed and were synthesized by TsingKe Biotech Co., Ltd. (Beijing, China) and then inserted into pMIR-Report Luciferase plasmid (miaolingbio, P0471,Wuhan, China), respectively, with empty pMIR-Report Luciferase plasmid (Luc-vec) as control. Internal control pRL-TK Renilla plasmid was generously provided by Dr. Pengsheng Zheng (Xi’an Jiaotong University, Xi’an, China). The reporter plasmids were co-transfected with pRL-TK Renilla plasmid into MC3T3-E1 cells, and miR-129-5p mimic/inhibitor transfection was performed as mentioned above. Luciferase reporter assays were performed with the dual-luciferase reporter assay system (Promega, E1910, Fitchburg, WI, United States) according to the manufacturer’s instruction. Luminescent signals were quantified, and each value from the firefly luciferase was normalized by Renilla luciferase.

### Therapeutic miR-129-5p Inhibitor in OVX Mice

To investigate therapeutic effect of miR-129-5p inhibitor on osteoporosis, 48 OVX mice were randomly divided into seven groups (baseline, sham, OVX, mock, inhibitor-NC, inhibitor-129-5p). The transfection was performed at 8 and 15 days after OVX, respectively. And mice were injected subcutaneously over the calvarial surface with inhibitor-129-5p or inhibitor-NC formulated with Entranster^TM^ *In vivo* Transfection Reagent twice per day, at the dosage of 40 μL (including 4.8 μg RNA) according to the manufacturer’s instructions. In the mock group, mice were injected with the same volume of normal saline mixed with transfection reagent. The mice in OVX group received no treatment. All mice were given the same standard diet during the experimental period. All mice of the baseline group and three mice from other groups were euthanized 11 days after OVX treatment, and calvarias were used for RT-PCR (*n* = 3/group). All the other mice were euthanized 35 days after OVX, and calvarias were used for immunohistochemical staining and histomorphometric analyses (*n* = 3/group; Yin et al., 2019).

### Manufacture and Transfection of Recombinant miR-129-5p Inhibitor

Recombinant miR-129-5p inhibitors were manufactured to achieve the translational medical application of miR-129-5p with high efficiency and low cost. The production was performed as previously described (Ho et al., 2018; Li X. et al., 2019). In brief, sequence of inhibitor-129-5p was designed and synthesized and then inserted into plasmid pBSMrnaSeph. The edited plasmid was transformed into *E. coli* (HST08). Total RNA of *E. coli* was extracted and purified by fast protein liquid chromatography. The purity of recombinant miR-129-5p inhibitors were checked by denaturing urea 8% PAGE. The whole manufacture process was performed by RQCON Biological Technology Co., Ltd. (Xi’an, China).

*In vitro* transfection of recombinant inhibitor-129-5p (nCAR/anti129) was performed by Lipofectamine^TM^ 2000 according to the manufacturer’s instructions (10 nM), using empty tRNA scaffold (tRNA^Met^ fused Sephadex aptamer, MSA) as control. As for *in vivo* transfection of recombinant miR-129-5p inhibitor, 18 OVX mice were randomly divided into five groups (baseline, sham, OVX, mock, MSA, nCAR/anti129). The transfection was performed at 8 and 15 days after OVX, respectively. Mice were injected subcutaneously over the calvarial surface with recombinant miR-129-5p inhibitor or MSA formulated with Entranster^TM^ *In vivo* Transfection Reagent twice per day, at the dosage of 40 μL (including 1.2 μg RNA) according to the manufacturer’s instructions. In the mock group, mice were injected with normal saline mixed with transfection reagent, and the OVX group was given no treatment. All mice received the same standard diet during the experimental period. The mice in the baseline group were sacrificed at 11 days after OVX treatment; all other mice were killed 35 days after OVX, and calvarias were processed for histomorphometric analyses (*n* = 3/group).

### Statistical Analysis

The statistical analyses of the data were performed with GraphPad Prism version 6.0 software (GraphPad Software, Inc., La Jolla, CA, United States), and a Student *t*-test was used. The data are presented as mean ± standard deviation (SD). *P* < 0.05 was considered statistically significant for all comparisons. Correlation between miR-129-5p and other genes was exhibited by linear regression and Pearson correlation coefficient.

## Data Availability Statement

The original contributions presented in the study are included in the article/Supplementary Material, further inquiries can be directed to the corresponding author.

## Ethics Statement

The animal study was reviewed and approved by the Institutional Experimental Animal Committee of Northwestern Polytechnical University, Xi’an, China.

## Author Contributions

CYi, YT, and AQ designed the experiments. CYi, YT, YY, CYa, PS, YZ, XW, KZ, JP, and DL performed the experiments and analyzed the data. CYi, YT, and YY wrote the manuscript. All authors read and approved the final manuscript.

## Conflict of Interest

The authors declare that the research was conducted in the absence of any commercial or financial relationships that could be construed as a potential conflict of interest.
