# Supplementary material for: miR-129-5p Inhibits Bone Formation Through TCF4
Source: Front Cell Dev Biol. 2020 Nov 6;8:600641. doi: 10.3389/fcell.2020.600641 (PMC7681249; doi:10.3389/fcell.2020.600641)
Supplement: Supplementary file 1 [file Data_Sheet_1.docx]

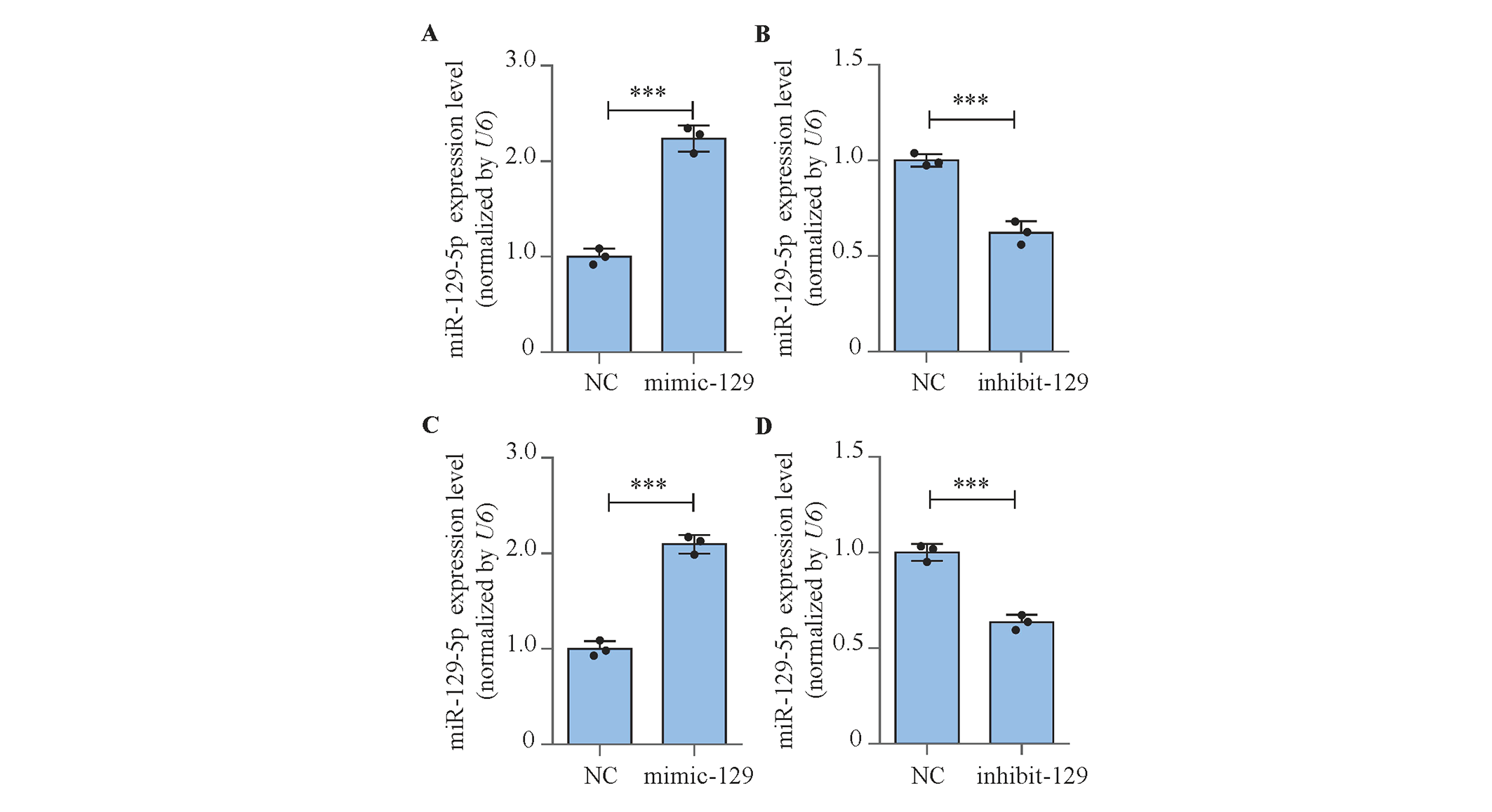


**Supplemental Figure 1. Effects of miR-129-5p mimic or inhibitor *in vitro* and *in vivo***

**A-B.** miR-129-5p expression levels of MC3T3-E1 cells treated with miR-129-5p mimic or inhibitor (compared to mimic- or inhibitor-NC respectively), as detected by RT-PCR (mean ± S.D., ****P*<0.001).

**C-D.** miR-129-5p expression levels in calvatia of C57BL/6 mice treated with mimic or inhibitor-129-5p (compared to mimic- or inhibitor-NC respectively), as detected by RT-PCR (mean ± S.D., ****P*<0.001).


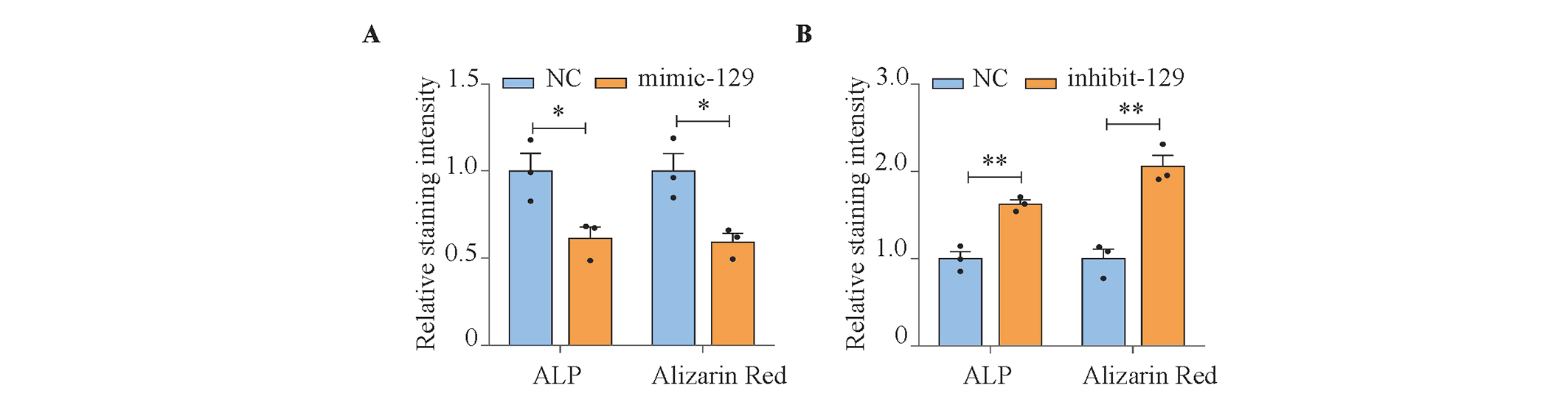


**Supplemental Figure 2. ALP and Alizarin Red staining intensities of MC3T3-E1 cells treated with miR-129-5p mimic or inhibitor**

**A.** ALP and Alizarin Red staining intensities of MC3T3-E1 cells treated with miR-129-5p mimic (mean ± S.D., **P*<0.05).

**B.** ALP and Alizarin Red staining intensities of MC3T3-E1 cells treated with miR-129-5p inhibitor (mean ± S.D., ***P*<0.01).


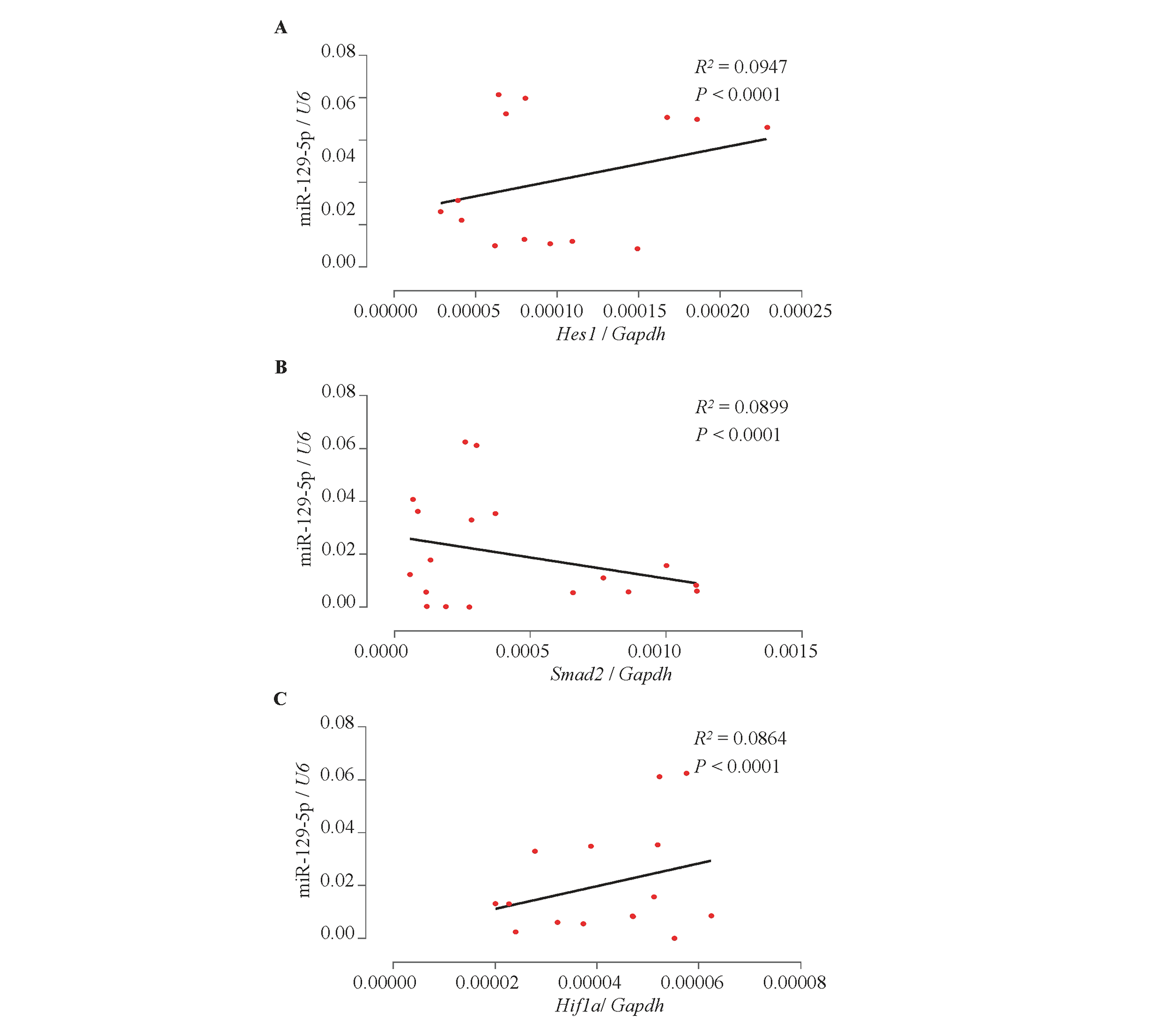


**Supplemental Figure 3. Correlation between miR-129-5p and osteogenic transcript factors**

**A-C.** Correlation analysis between miR-129-5p and *Hes1*, *Smad2* and *Hif1α* mRNA levels in femur tissues from C57BL/6 mice respectively, as detected by RT-PCR.


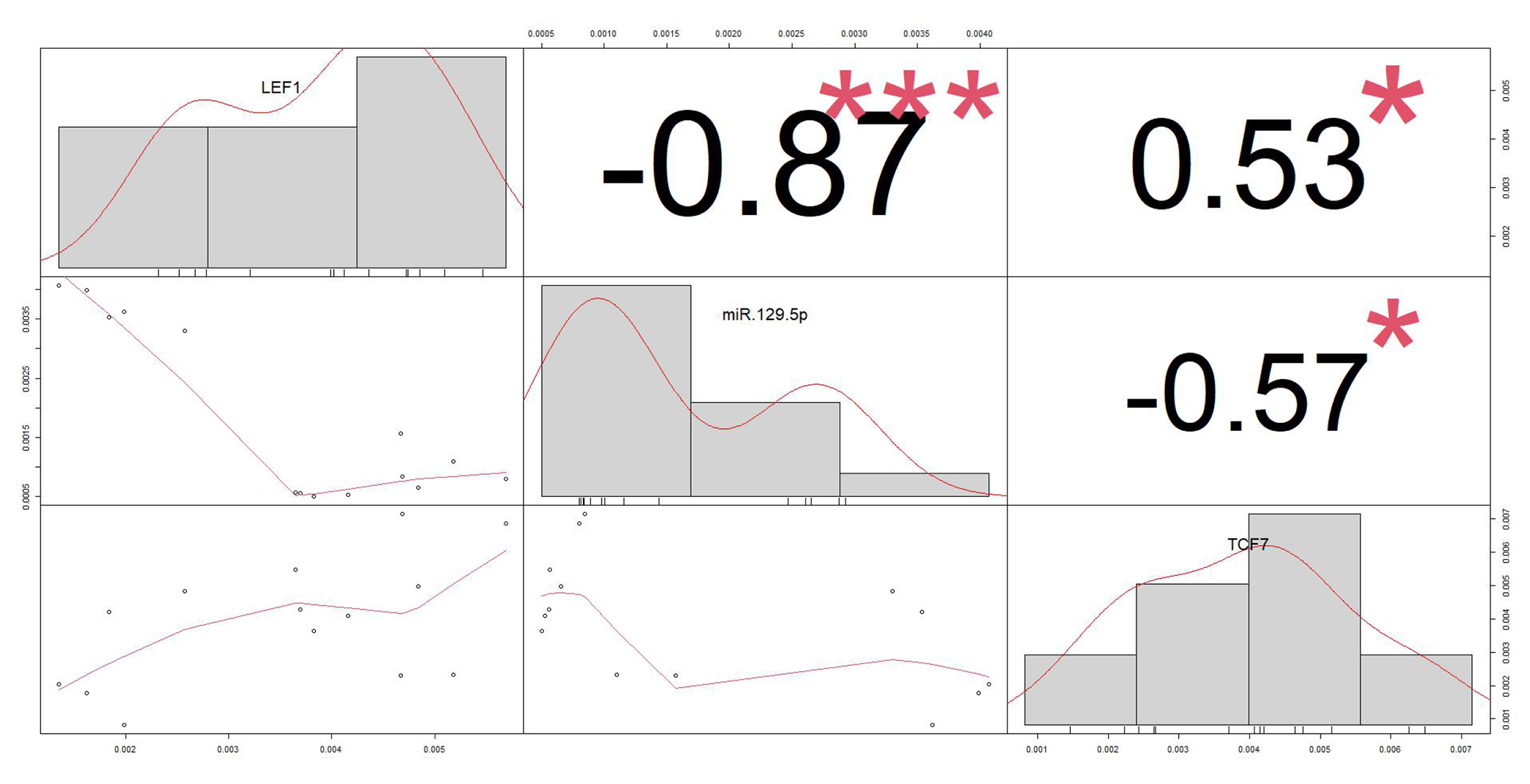


**Supplemental Figure 4. Pearson correlation coefficient between miR-129-5p, *Tcf7*, and *Lef1***

**
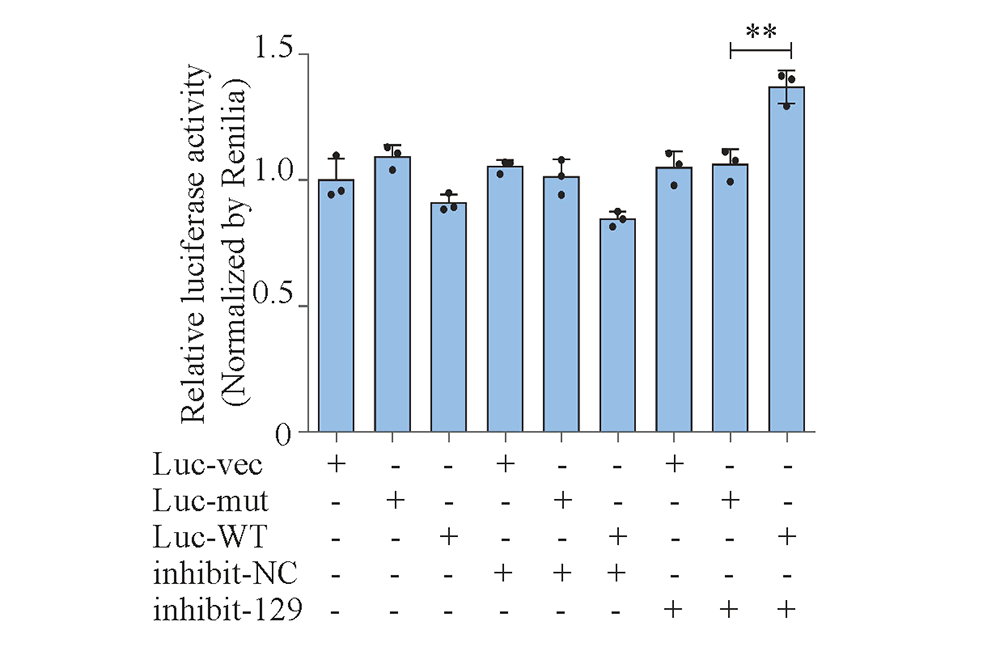
**

**Supplemental Figure 5. Bonding effect of miR-129-5p and *Tcf4-*3′UTR, as detected by luciferase reporter assay.**


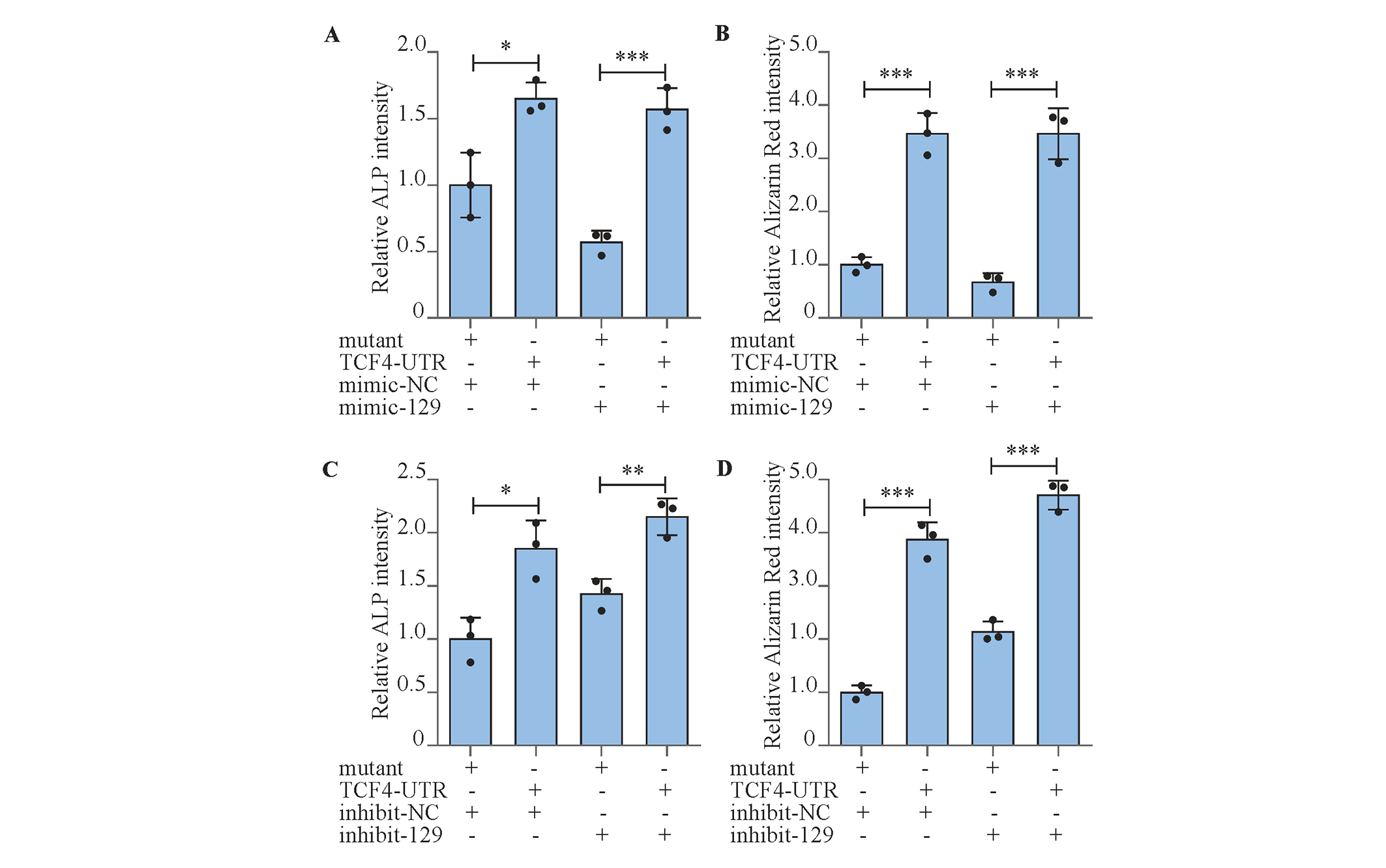


**Supplemental Figure 6. ALP and Alizarin Red staining intensities of MC3T3-E1 cells treated with miR-129-5p mimic or inhibitor**

**A-B.** ALP and Alizarin Red staining intensities of MC3T3-E1 cells treated with *Tcf4-*3′UTR plasmid and mimic-129-5p (mean ± S.D., **P*<0.05, ****P*<0.001). Mutant/TCF4-UTR: expression plasmid containing mutant/wild-type miR-129-5p binding site sequence of *Tcf4-*3′UTR.

**C-D.** ALP and Alizarin Red staining intensities of MC3T3-E1 cells treated with *Tcf4-*3′UTR plasmid and inhibitor-129-5p (mean ± S.D., **P*<0.05, ***P*<0.01, ****P*<0.001).

**
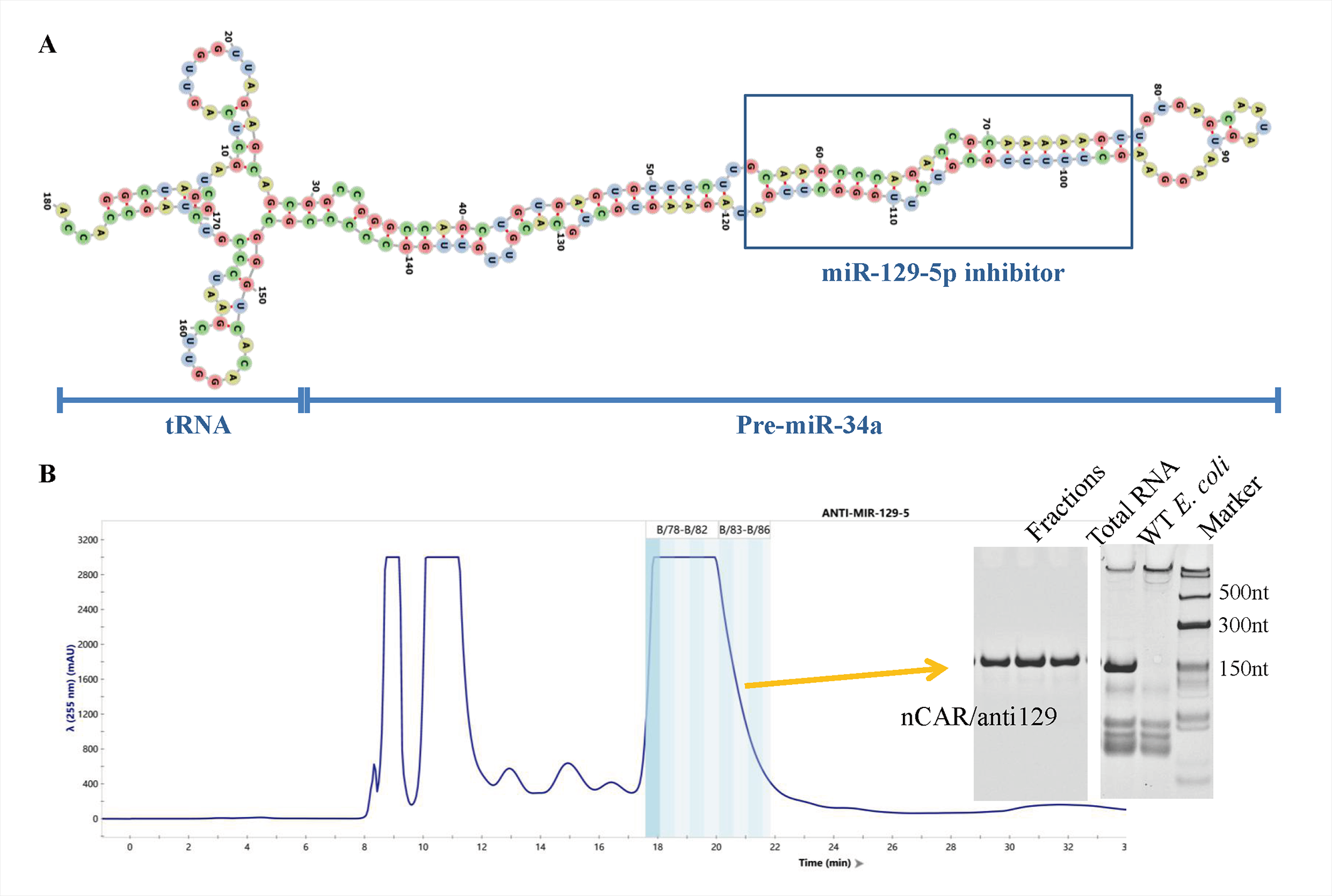
**

**Supplemental Figure 7. Bioengineering of recombinant miR-129-5p inhibitor.**

**A.** The secondary structure of bioengineered recombinant miR-129-5p inhibitor (nCAR/anti-129) was predicted by RNAfold (<http://rna.tbi.univie.ac.at/cgi-bin/RNAWebSuite/RNAfold.cgi>),where the miR-129-5p inhibitor sequence replaced miR-34a duplexes within the tRNA/pre-miR-34A-based non-coding RNA carrier (nCAR).

**B.** Representative FPLC trace during the purification of nCAR/anti129.The insert is urea-PAGE analysis of the targeted nCAR/anti129 expression in HST08 *E. coli* and the collected RNA fractions eluted at 17.6 min.


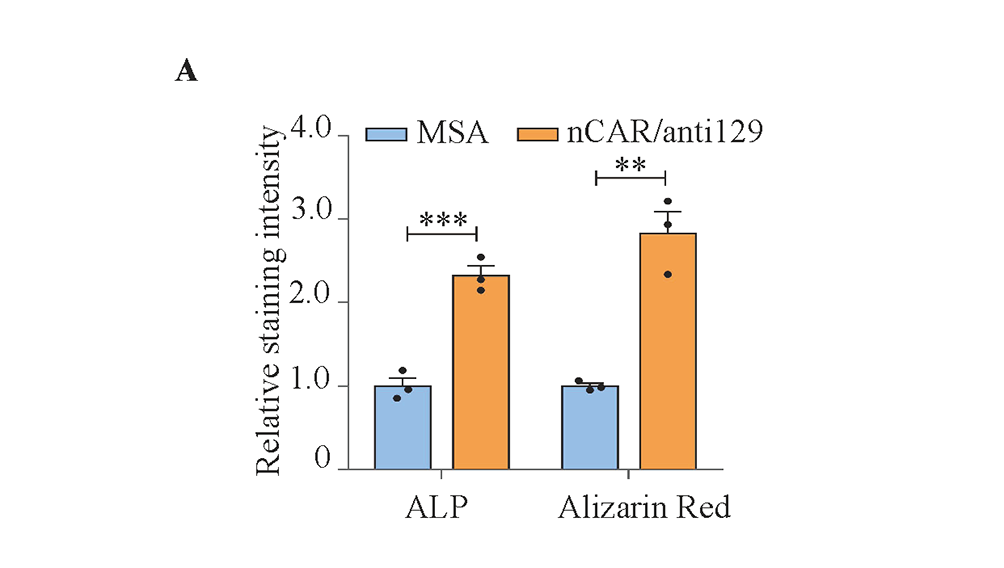


**Supplemental Figure 8. ALP and Alizarin Red staining intensities of MC3T3-E1 cells treated with recombinant miR-129-5p inhibitor**

**
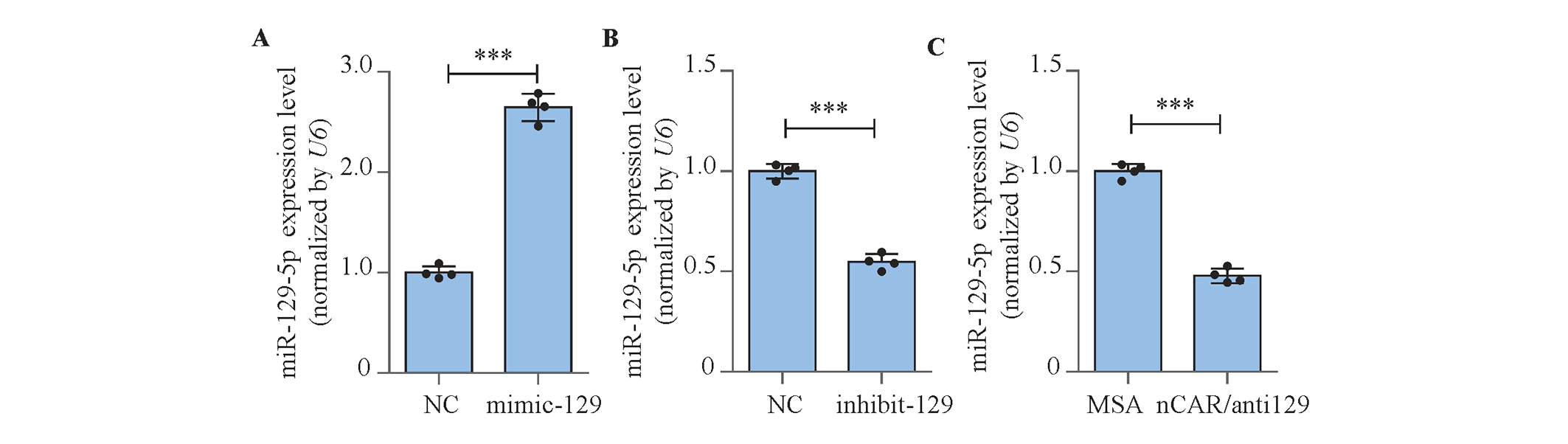
**

**Supplemental Figure 9. Effects of miR-129-5p mimic or inhibitor in mesenchymal stem cell**

**A-B.** miR-129-5p expression levels of C3H10 T1/2 cells treated with miR-129-5p mimic or inhibitor (compared to mimic- or inhibitor-NC respectively), as detected by RT-PCR (mean ± S.D., ****P*<0.001).

**C.** miR-129-5p expression levels of C3H10 T1/2 cells treated with recombinant miR-129-5p inhibitor, as detected by RT-PCR (mean ± S.D., ****P*<0.001). MSA: tRNA^Met^ fused Sephadex aptamer. nCAR/anti129: novel recombinant miR-129-5p inhibitor.

**
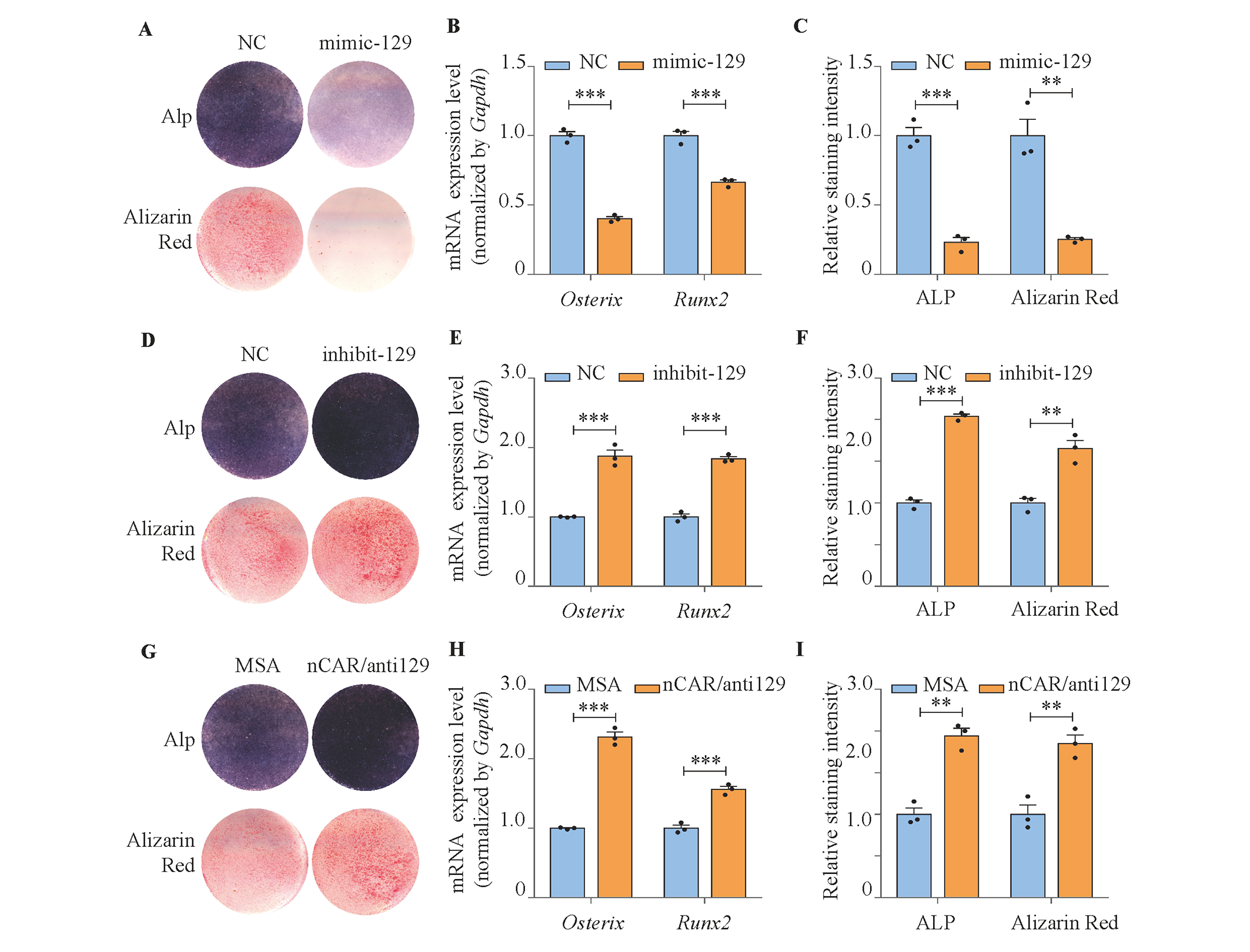
**

**Supplemental Figure 10. miR-129-5p inhibited osteoblast differentiation in mesenchymal stem cell**

**A.** Alp and Alizarin Red staining of C3H10 T1/2 cells treated with mimic-129-5p (compared to mimic-NC), as detected by Alp staining and Alizarin Red staining. Alp: results of Alp staining. Alizarin Red: results of Alizarin Red staining. NC: mimic-NC. mimic-129: mimic-129-5p.

**B.** *Osterix* and *Runx2* expression levels of C3H10 T1/2 cells treated with mimic-129-5p, as detected by RT-PCR (mean ± S.D., ****P*<0.001).

**C.** ALP and Alizarin Red staining intensities of C3H10 T1/2 cells treated with miR-129-5p mimic (mean ± S.D., ***P*<0.01, ****P*<0.001).

**D.** Alp and Alizarin Red staining of C3H10 T1/2 cells treated with inhibitor-129-5p (compared to inhibitor-NC), as detected by Alp staining and Alizarin Red staining. NC: inhibitor-NC. inhibit-129: inhibitor-129-5p.

**E.** *Osterix* and *Runx2* expression levels of C3H10 T1/2 cells treated with inhibitor-129-5p, as detected by RT-PCR (mean ± S.D., ****P*<0.001).

**F.** ALP and Alizarin Red staining intensities of C3H10 T1/2 cells treated with miR-129-5p inhibitor (mean ± S.D., ***P*<0.01, ****P*<0.001).

**G.** Alp and Alizarin Red staining of C3H10 T1/2 cells treated with treated with recombinant miR-129-5p inhibitor, as detected by Alp staining and Alizarin Red staining. MSA: tRNA^Met^ fused Sephadex aptamer. nCAR/anti129: novel recombinant miR-129-5p inhibitor.

**H.** *Osterix* and *Runx2* expression levels of C3H10 T1/2 cells treated with recombinant miR-129-5p inhibitor, as detected by RT-PCR (mean ± S.D., ****P*<0.001).

**I.** ALP and Alizarin Red staining intensities of C3H10 T1/2 cells treated with with recombinant miR-129-5p inhibitor (mean ± S.D., ***P*<0.01).


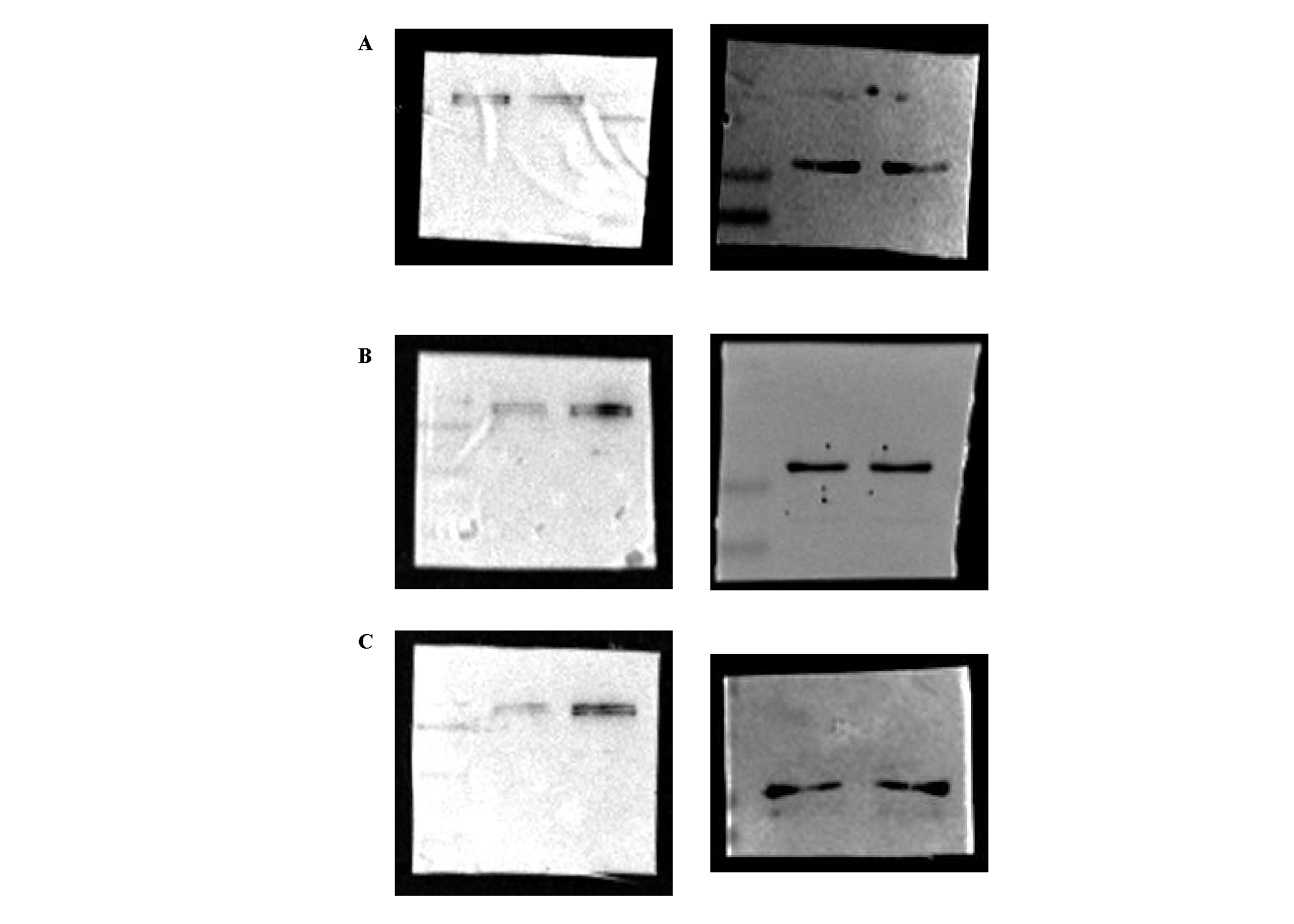


**Supplemental Figure 11. Original scaned images for western blot**

**A.** Original scaned images for western blot in figure 4A. Left: TCF4. Right: GAPDH.

**B.** Original scaned images for western blot in figure 4B. Left: TCF4. Right: GAPDH.

**C.** Original scaned images for western blot in figure 8G. Left: TCF4. Right: GAPDH.
